# Glycoside Phosphorylase Catalyzed Cellulose and β-1,3-Glucan Synthesis Using Chromophoric Glycosyl Acceptors

**DOI:** 10.1021/acs.biomac.4c00455

**Published:** 2024-07-18

**Authors:** Robert Pylkkänen, Hannu Maaheimo, Ville Liljeström, Pezhman Mohammadi, Merja Penttilä

**Affiliations:** † VTT Technical Research Centre of Finland Ltd., FI-02044 VTT, Finland; ‡ Department of Bioproducts and Biosystems, School of Chemical Engineering, Aalto University, FI-00076 AALTO, Finland; § Nanomicroscopy Center, OtaNano, Aalto University, FI-00076 AALTO, Finland

## Abstract

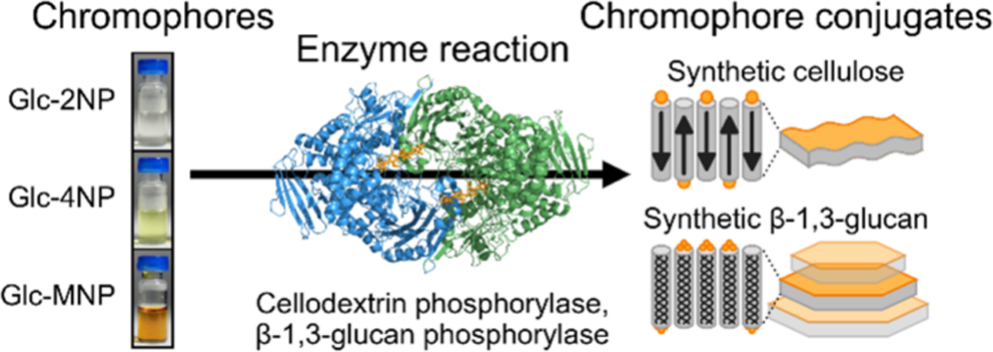

Glycoside phosphorylases are enzymes that are frequently used for polysaccharide synthesis. Some of these enzymes have broad substrate specificity, enabling the synthesis of reducing-end-functionalized glucan chains. Here, we explore the potential of glycoside phosphorylases in synthesizing chromophore-conjugated polysaccharides using commercially available chromophoric model compounds as glycosyl acceptors. Specifically, we report cellulose and β-1,3-glucan synthesis using 2-nitrophenyl β-d-glucopyranoside, 4-nitrophenyl β-d-glucopyranoside, and 2-methoxy-4-(2-nitrovinyl)phenyl β-d-glucopyranoside with *Clostridium thermocellum* cellodextrin phosphorylase and *Thermosipho africanus* β-1,3-glucan phosphorylase as catalysts. We demonstrate activity for both enzymes with all assayed chromophoric acceptors and report the crystallization-driven precipitation and detailed structural characterization of the synthesized polysaccharides, i.e., their molar mass distributions and various structural parameters, such as morphology, fibril diameter, lamellar thickness, and crystal form. Our results provide insights for the studies of chromophore-conjugated low molecular weight polysaccharides, glycoside phosphorylases, and the hierarchical assembly of crystalline cellulose and β-1,3-glucan.

## Introduction

Colorants, i.e., dyes and pigments, are used in a wide range of different industries in textiles, plastics, coatings, inks, and cosmetics. Synthetic colorants, which are mostly derived from petrochemicals, have predominated in the colorant market due to low manufacturing cost, wide range of available colors, ease of application, and high stability.^1^ Recently, however, there has been growing interest in natural colorants, which has been driven by consumer demand and regulatory pressure, as well as growing concerns about the safety and environmental impact of synthetic dyes.^2−7^

Natural colorants, which are derived from plants, animals, and minerals, have been used for thousands of years^8^ to add color to a variety of products, such as food, clothing, and cosmetics. However, natural colorants also suffer from several sustainability issues despite being obtainable from renewable resources. For example, their production is typically seasonal and expensive, and the colorants themselves are often impure, unstable, and/or require mordants as fixatives.^9^ Interestingly, colorant production with microbes could potentially solve many of these issues because microbes can be grown on a large scale on cheap substrates under controlled conditions in a sustainable manner. In this context, several biotechnological approaches have been recently developed that have enabled, for example, the production of carminic acid, a widely used red natural colorant found in scale insects, using genetically engineered microbes.^10−12^

Biotechnology enables not only new ways to produce natural colorants but also new ways to modify them for more desirable physicochemical properties. For example, glycosyl groups can be introduced into chromophoric molecules using glycosyltransferase enzymes.^13−15^ Interestingly, the glycosyl moieties of glycosides can also be used as glycosyl acceptors for glycan chain synthesis using phosphorylase enzymes.^16^ Because polysaccharides form intermolecular interactions via hydrogen bonding and van der Waals forces, a short polysaccharide chain attached to a chromophore could enable improved binding on polysaccharide-based textiles, such as cotton, reducing the need for mordants.^17^ Moreover, a covalent linkage between a chromophore and a short polysaccharide chain could even offer new ways of covalently linking chromophores to longer polysaccharides, e.g., to cellulose chains in cotton using endotransglucosylase/hydrolase enzymes.^18^

Glycoside phosphorylases are a class of carbohydrate-active soluble enzymes that are frequently utilized for poly- and oligosaccharide synthesis, with a broad substrate specificity toward non-native acceptor molecules.^16,19−22^ They utilize simple sugar phosphate donors and catalyze the reversible formation and cleavage of glycosidic linkages with strict regioselectivity,^23^ which makes them desirable candidates in the synthesis of various carbohydrate structures. The process can also be scaled up to relatively large scales; for example, the commercial synthesis of 2-*O*-α-d-glucosylglycerol^24^ and kilogram scale synthesis of lacto-*N*-biose^25^ have been successfully carried out using phosphorylases. More recently, cellodextrin phosphorylase enzymes have been used to synthesize reducing-end-modified synthetic cellulose chains, which has been made possible due to the relaxed substrate specificity of these enzymes. Some examples of achieved functionalizations include the introduction of alkyl,^26,27^ amino,^28^ azide,^29^ azido,^30^ carboxyl,^31^ fluorine,^32^ phenol,^33^ thiol,^34^ and vinyl^19^ groups and can be found reviewed elsewhere.^35,36^ The synthesis of reducing-end-functionalized crystalline β-1,3-glucans has received far less attention and has not been reported using glycoside phosphorylases to our knowledge.

In this study, we report the synthesis and structural characterization of enzymatically synthesized chromophore-conjugated cellulose and β-1,3-glucan. While we have utilized synthetic nitrophenyl glycosides as model compounds in this study, our approach underscores the potential of enzymatic synthesis to advance the field of chromophore-conjugated polysaccharides. The use of synthetic chromophores in our research serves as a proof of concept, demonstrating the feasibility and efficiency of utilizing chromophore-conjugated glycosides as acceptors for enzymatic synthesis. However, our goal is to transition toward more sustainable and environmentally friendly natural colorants. For enzymatic synthesis, we employed *Clostridium thermocellum* cellodextrin phosphorylase (*Ct*CDP)^37−39^ and *Thermosipho africanus* β-1,3-glucan phosphorylase (*Ta*1,3BGP)^40−42^ mediated oligomerization of the glucose 1-phosphate donor (Glc-1P) in the presence of glucose (Glc) or commercially available chromophoric β-glycoside acceptors: 2-nitrophenyl β-d-glucopyranoside (Glc-2NP), 4-nitrophenyl β-d-glucopyranoside (Glc-4NP), and 2-methoxy-4-(2-nitrovinyl)phenyl β-d-glucopyranoside (Glc-MNP). We could measure high glucose 1-phosphate conversion rates with all of the acceptors mentioned earlier, suggesting that both of the employed enzymes are promising candidates for the synthesis of chromophoric carbohydrate structures using non-native acceptors. We also report the crystallization-driven precipitation and detailed structural characterization of the synthesized polysaccharides, i.e., their molar mass distributions and various structural parameters, such as morphology, fibril diameter, lamellar thickness, and crystal form. The employed strategies could be extended to a wide range of different chromophoric glycosides to generate polysaccharides with covalently linked chromophores.

## Experimental Section

### Materials

α-d-glucose 1-phosphate disodium salt hydrate (Glc-1P, ≥97%), 2-nitrophenyl β-d-glucopyranoside (Glc-2NP, ≥99%), *p*-nitrophenyl β-d-glucopyranoside (Glc-4NP, ≥98%), 2-methoxy-4-(2-nitrovinyl)phenyl β-d-glucopyranoside (Glc-MNP, ≥95%), 4-nitrophenyl α-d-maltohexaoside (≥98%), 4-(2-hydroxyethyl)-1-piperazineethanesulfonic acid (HEPES, ≥99.5%), and sodium deuteroxide solution (30 wt % in D_2_O, 99 atom % D) were purchased from Sigma-Aldrich and used as received. d-(+)-Glucose, AnalaR NORMAPUR analytical reagent (Glc, 101176K) was purchased from VWR.

### Cloning, Expression, and Purification of *Ct*CDP and *Ta*1,3BGP

The nucleotide sequence of the cellodextrin phosphorylase gene from *C. thermocellum* (*cdp*) was synthesized and codon-optimized for expression in the yeast *Saccharomyces cerevisiae* by Thermo Fisher Scientific (Espoo, Finland). Putative N-glycosylation sites were eliminated by replacing Ser and Thr residues from the consensus sequence Asn-X-Ser/Thr with Ala. The β-1,3-glucan phosphorylase gene (*bgp*) from *T. africanus* was synthesized and codon-optimized for expression in the yeast *S. cerevisiae* by Eurofins Genomics (Espoo, Finland). For protein purification, a 6× His-tag was included in the N-terminal end of the *cdp* and *bgp* genes.

Both genes were cloned into a yeast expression vector under the regulation of the constitutive *ENO1* promoter and *ENO1* terminator by using yeast homologous recombination. Both of the resulting plasmids (pRPC-008 and pRPC-041 for *Ct*CDP and *Ta*1,3BGP, respectively) contained the *URA3* selection marker and 2 μm origin of replication for autonomous replication in yeast. Plasmids were transformed into *Escherichia coli* XL1-Blue cells for replication, and the correct plasmids were confirmed with sequencing and transformed separately into *S. cerevisiae* Inv*Sc*1 strain (genotype: *MATa*, *his3*Δ*1*, *leu2*, *trp1-289*, *ura3-52/MATα*, *his3*Δ*1*, *leu2*, *trp1-289*, and *ura3-52*) using the lithium acetate method.^43^ Transformants were selected for uracil prototrophy on SCD^–URA^ (20 g/L glucose and 6.7 g/L yeast nitrogen base supplemented with appropriate amino acids).

For protein expression, yeast strains harboring the yeast expression vectors were grown in 500 mL of SCD^–URA^ medium in 2.5 L Erlenmeyer flasks (for a total volume of 3 L) at 30 °C and 200 rpm. Cells were harvested by centrifugation at 2700*g* for 15 min and resuspended in ice-cold lysis buffer (50 mM Tris-HCl, 500 mM NaCl, pH 7.4), supplemented with protease inhibitors (1× cOmplete, ethylenediaminetetraacetic acid (EDTA)-free Protease Inhibitor Cocktail, Roche). Lysis was performed with three passes through a French press at 10,000 psi.

For protein purification, cell lysates were centrifuged at 27,000*g* for 45 min at 4 °C, and the supernatant was loaded on a 5 mL HiTrap Chelating HP column equilibrated with 50 mM Tris-HCl and 500 mM NaCl, pH 7.4. Both enzymes were eluted with a 30 mL gradient from 0 to 500 mL imidazole, and fractions containing *Ct*CDP or *Ta*1,3BGP (analyzed by sodium dodecyl-sulfate polyacrylamide gel electrophoresis (SDS-PAGE)) were pooled and concentrated using Vivaspin 20, a 10 kDa MWCO PES ultrafiltration device (GE Healthcare). During the concentration, the buffer was exchanged to 200 mM HEPES–NaOH, pH 7.0, with 1 mM dithiothreitol (DTT). Protein concentration was estimated with the Bio-Rad Protein Assay using the manufacturer’s standard procedure for microtiter plates.

### Glucan Synthesis

All enzymatic reactions were carried out in 200 mM 2-[4-(2-hydroxyethyl)piperazin-1-yl]ethanesulfonic acid (HEPES)–NaOH buffer, pH 7.0, with 1 mM DTT at 50 °C. The glycosyl donor (Glc-1P) concentration was 200 mM, and the glycosyl acceptor (Glc, Glc-2NP, Glc-4NP, Glc-MNP) concentration was 50 mM. Glc-MNP was incubated for 5 min at 95 °C for solubilization. Concentrations of *Ct*CDP and *Ta*1,3BGP were 180 and 300 nM, respectively. After the reactions were finished, insoluble fractions were separated by centrifugation and washed several times in DDIW.

### Phosphate Release

Phosphate release was measured using a Malachite Green Phosphate Assay Kit (BioAssay Systems) according to the manufacturer’s instructions. Briefly, 80 μL of the diluted (1:5000 in DDIW) reaction supernatant was mixed with 20 μL of malachite green reagent in a 96-well plate. The plate was incubated at room temperature for 45 min, and the released phosphate was quantified by measuring and comparing absorbance at 620 nm against a standard curve of known inorganic phosphate concentrations.

To quantify reaction rates, the experimental data was fitted to a first-order reaction model, which describes the liberation of phosphate over time. The model is given by the following equation:
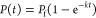


where *P*(t) is the concentration of phosphate at time *t*, *P*_f_ is the final phosphate concentration, *k* is the first-order rate constant, and *t* is the reaction time.

This equation assumes that *P*_f_ represents the final phosphate concentration when all substrates have been converted, simplifying the fitting process by not taking into account product inhibition or other factors. Data fitting was done using the curve_fit function from the scipy.optimize module in Python. The covariance matrix of the parameter estimates was obtained as part of the fitting process and was used to calculate the standard deviations of the fitted parameters as the square root of the diagonal elements of the covariance matrix. For this approach, the experimental data was normalized so that values of *P*(t) are positive, as negative values distort the fitting process.

To compare different reaction rates and assess their statistical significance, the *t*-statistic and the corresponding *p*-value were calculated using the independent two-sample *t-*test using ttest_ind_from_stats from the scipy.stats module in Python.

### Size-Exclusion Chromatography (SEC)

Reaction products were centrifuged and washed several times, freeze-dried overnight, and dissolved in 1 M NaOH. Relative molar masses were determined using size-exclusion chromatography in 0.1 M NaOH eluent with PSS MCX 1000 and 100,000 Å columns and a Waters 2414 Refractive Index Detector (Waters). The differential molar mass distributions were calculated against pullulan standards (Shodex, Germany).

### Matrix-Assisted Laser Desorption Ionization Time-of-Flight Mass Spectrometry (MALDI-ToF MS)

Reaction products were washed, centrifuged several times, and resuspended in DDIW. One microliter of aqueous solutions was mixed in a polymerase chain reaction (PCR) tube with 1 μL of 10 mg/mL 2,5-dihydroxybenzoic acid in 50% acetonitrile and spotted directly on a MALDI target. MALDI-ToF spectra were recorded on an ultrafleXtreme III MALDI-ToF/ToF instrument (Bruker Daltonics, Germany) and calibrated using either Protein Calibration Standard I or Protein Calibration Standard II (Bruker Daltonics, Germany).

### NMR Spectroscopy

Reaction products were washed and centrifuged several times and freeze-dried overnight, and 5–10 mg of samples was dissolved in 220 μL of 4% (w/w) NaOD-D_2_O. Three millimeter NMR tubes were used in order to keep the 90° pulses reasonably short (∼13 μs for ^1^H) despite the high salt concentration.

The NMR spectra were acquired at 22 °C on a 600 MHz Bruker Avance III NMR spectrometer equipped with a cryogenically cooled 5 mm QCI (H-P,C,N-D) probehead. The one-dimensional (1D) ^1^H spectra were acquired using a 4 s volume selective presaturation of the residual water signal (so-called 1D nuclear Overhauser effect spectroscopy (NOESY) presaturation). Two-dimensional (2D) experiments correlated spectroscopy (COSY), total correlation spectroscopy (TOCSY), multiplicity edited heteronuclear single quantum coherence spectroscopy (HSQC), and heteronuclear multiple bond correlation (HMBC) were carried out using standard Bruker pulse programs *cosygpppqf, dipsi2gpphpr, hsqcedetgpsisp2.2*, and *hmbcetgpl3nd*, respectively. In TOCSY, the mixing time was 120 ms, and the long-range coupling constant in HMBC was 8 Hz. In addition, pure shift HSQC (*hsqcetgpsp.2_bbhd*)^44^ was used for better resolution and accurate determination of chemical shifts.

### Scanning Electron Microscopy (SEM)

Reaction products were washed and centrifuged several times and resuspended in DDIW. Thirty microliters of the aqueous solution was plunged into propane (−180 °C) to preserve internal structures. Samples were then handled in liquid nitrogen and freeze-dried overnight. Imaging was performed with a Zeiss FE SEM field emission microscope with a variable pressure operating at 1.5 kV. A thin platinum–palladium coating was sputtered onto the sample surface prior to imaging.

### Small- and Wide-Angle X-Ray Scattering (SAXS and WAXS)

Both wet and dry samples were characterized by SAXS and WAXS. Wet samples were sealed using hot glue in borosilicate Mark tubes (diameter 1.5 mm, wall thickness 10 μm, Hilgenberg GmbH, Germany), and dry samples were sealed between Kapton tape during the experiment.

SAXS/WAXS measurements were taken using a Xenocs Xeuss 3.0 SAXS/WAXS system (Xenocs SAS, France). The system includes a microfocus X-ray source (sealed tube) with a Cu target and a multilayer mirror, which yields a parallel beam with a nominal wavelength of 1.542 Å (combined Cu Kα_1_ and Cu Kα_2_ characteristic radiation). The source operates at 50 kV and 0.6 mA. The beam is collimated by a set of variable slits, and the beam size at the sample was 0.7 mm during the experiments. The system does not include a beam stop, which enables the direct measurement of sample transmission. The data was acquired using a 2D area detector (Eiger2 R 1M, Switzerland) that was in the evacuated chamber. The 1D data shown in this article are the azimuthal average of the 2D data. The scattering contributions (background scattering) measured from an empty capillary and pure buffer (water) were subtracted from the data according to the measured transmission. For some samples, the scattering contribution from water needed to be scaled by max. ±15% to compensate for the variation of capillary diameter and water content. The sample-to-detector distance (1100 mm for SAXS and 55 mm for WAXS) was calibrated by measuring the diffraction from a known LaB6 standard sample.

Model fitting was done as described elsewhere.^40^ Briefly, a paracrystal lamellar model^45^ was employed, and data was fitted to the model using the curve_fit function of the SciPy 1.7.3 package in Python 3.8.16. Background (water) was subtracted from the measurements prior to fitting.

## Results and Discussion

**Figure 1:**
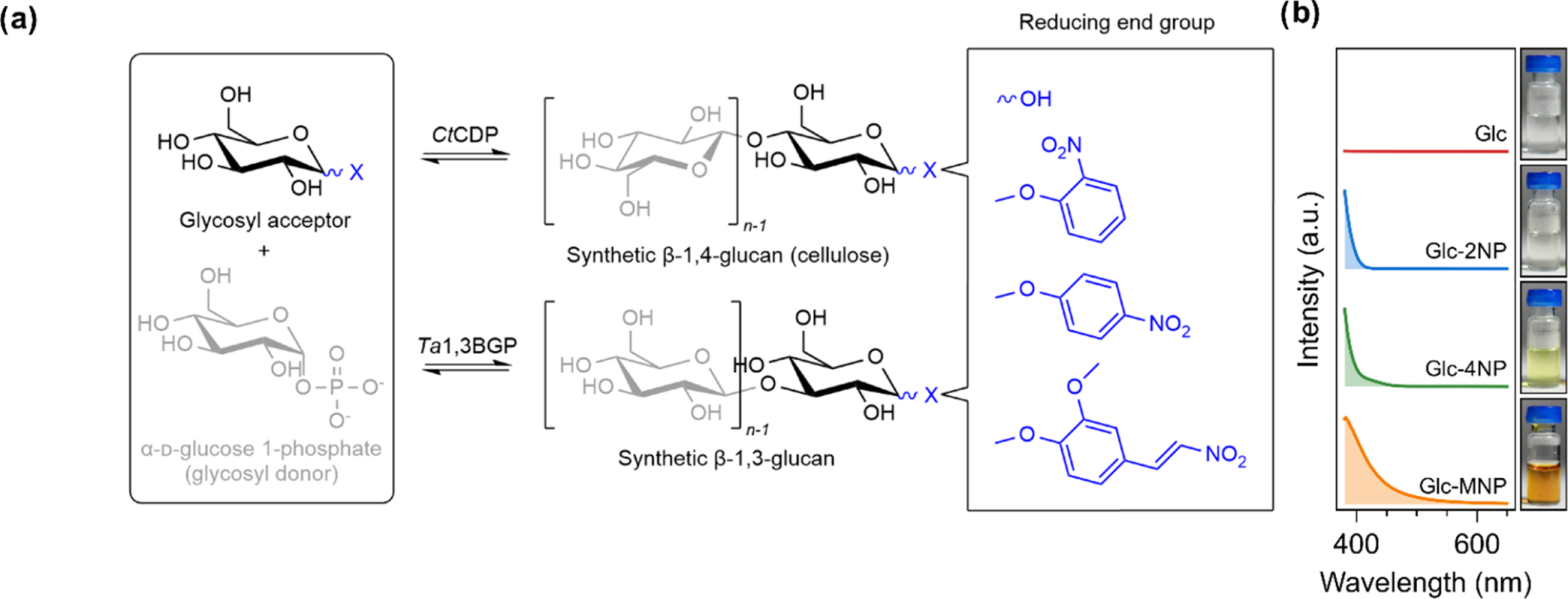
Enzymatic synthesis reactions for cellulose synthesis using *Ct*CDP and β-1,3-glucan synthesis using Ta1,3BGP and the visual characteristics of the four glycosyl acceptors used in this study. (a) Reaction scheme depicting the synthesis of reducing end-modified cellulose and β-1,3-glucan. The release of *n*-1 free phosphate molecules during synthesis is omitted for the sake of clarity. (b) Absorbance spectra of the glycosyl acceptors in the visible light spectral region and photographs of the reaction mixtures before the reactions were started by the addition of the enzyme.

In order to determine whether the chosen chromophoric glycosides, i.e., Glc-2NP, Glc-4NP, or Glc-MNP, are suitable acceptors for cellulose or β-1,3-glucan synthesis using *Ct*CDP or *Ta*1,3BGP, respectively, we performed cellulose and β-1,3-glucan synthesis reactions with the respective enzymes and one of the three acceptors or glucose (Glc) as a control (Figure 1a). The employed glycosyl donor and acceptor concentrations (200 and 50 mM, respectively), as well as pH 7.0, were chosen based on our previous results and are typical for synthesis reactions with phosphorolytic enzymes.^38,40^ Glucose was chosen as the control glycosyl acceptor for these reactions because it is a suitable acceptor for both *Ct*CDP and *Ta*1,3BGP. All enzymatic synthesis reactions were performed at 50 °C because this temperature has been reported to be optimal for β-1,3-glucan microparticle formation using *Ta*1,3BGP^40^ and cellulose nanosheet formation using *Ct*CDP.^46^ It should be noted that the optimum temperature in terms of relative enzymatic activity has been reported as 60 °C for *Ct*CDP^47^ and 75 °C for *Ta*1,3BGP,^48^ but here, we specifically refer to the optimum temperature in terms of obtaining the desired polysaccharide structures. Before enzymatic reactions were started, we characterized the visual appearances of the chromophoric β-glycosides in the soluble form at 50 mM concentration by quantifying the absorbance spectra of the solutions in the visible light spectrum, i.e., 380–750 nm, and by photographing them (Figure 1b). Glc and Glc-2NP appeared colorless despite Glc-2NP showing absorption toward the shorter wavelengths of the visible spectrum. The solution containing Glc-4NP was slightly yellow, while Glc-MNP was brown. It should be noted that, for example, 4-nitrophenol has been reported to be colorless below pH 5.4 and yellow above pH 7.5. The yellow color is associated with the 4-nitrophenolate form, which has its absorbance maximum at 405 nm.^49^

### Enzymatic Synthesis

**Figure 2:**
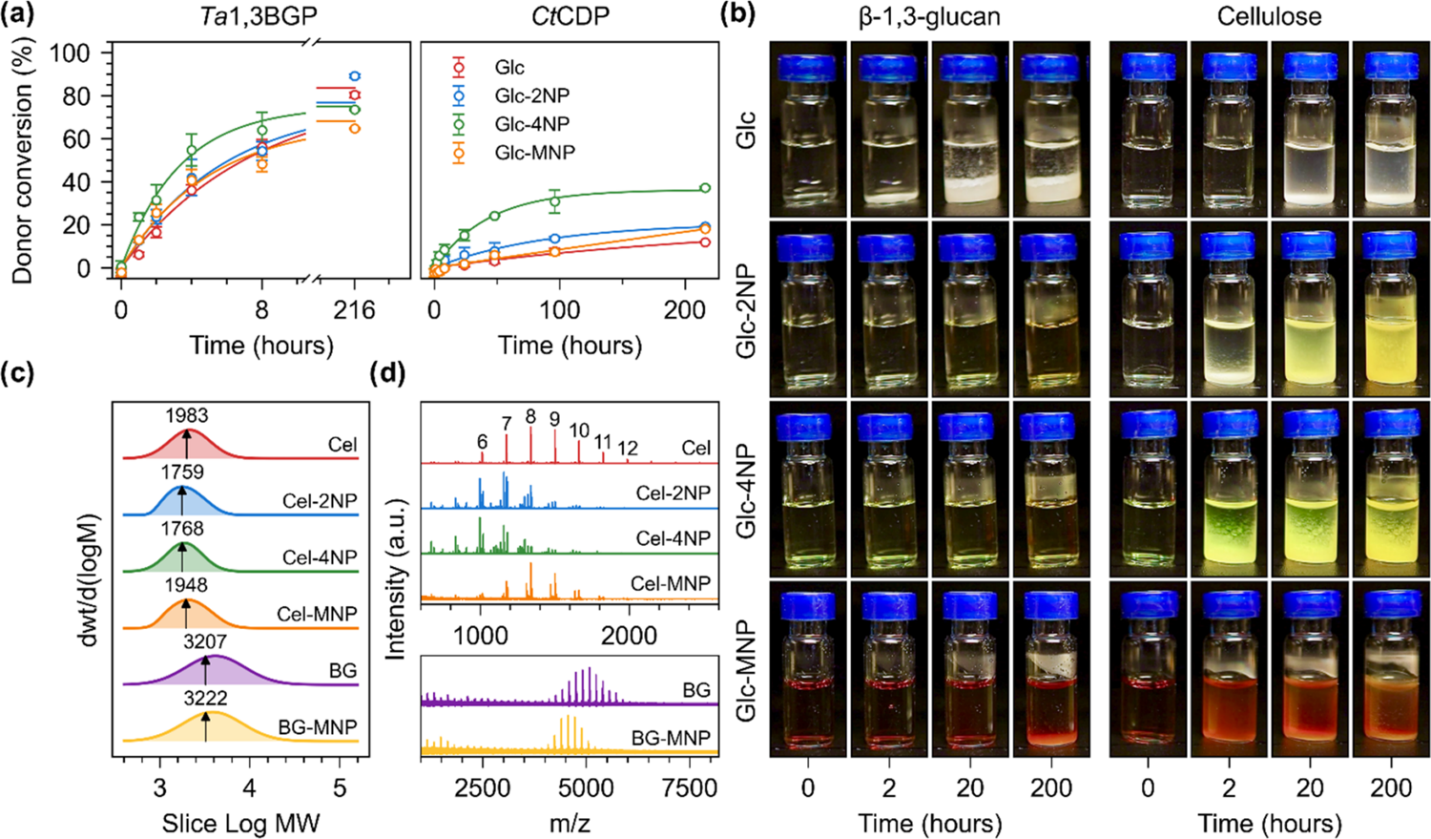
Characterization of enzymatic synthesis reactions and molar mass distributions of produced insoluble cellulose and β-1,3-glucan. (a) Donor conversion (%) as a function of time quantified by measuring the amount of free orthophosphate liberated from glucose 1-phosphate using a colorimetric assay and corresponding fit to first-order kinetics equation. (b) Photographs showing the formation of insoluble precipitate over time during the enzymatic synthesis reactions. (c) Molar mass distributions (MMDs) of the insoluble reaction products (cellulose, cellulose-2NP, cellulose-4NP, β-1,3-glucan, and β-1,3-glucan-MNP) measured using size-exclusion chromatography (SEC). *M*_n_ (number-average molar mass) of each distribution is indicated by an arrow along with its value (g/mol). (d) MMDs of the insoluble reaction products measured using MALDI-ToF-MS. The number of glucose units per peak is indicated above each peak for cellulose synthesized using Glc as the acceptor.

Enzymatic synthesis reactions were started by adding either *Ta*1,3BGP or *Ct*CDP. To determine the progression of the reactions during synthesis, we quantified the amount of free inorganic phosphate liberated due to the conversion of the glycosyl donor, Glc-1P (Figure 2a, full range of experimental data together with curves obtained from fitting to the first-order kinetics equation in Figure S1). Donor conversion could be measured with both enzymes and all acceptors. To be more specific, the first-order rate constant *k* obtained by fitting a first-order model to the experimental data were 0.010 ± 0.002, 0.017 ± 0.004, 0.028 ± 0.005, and 0.005 ± 0.002 h^–1^ for *Ct*CDP-catalyzed synthesis reactions using Glc, Glc-2NP, Glc-4NP, or Glc-MNP as the acceptor, respectively. For *Ta*1,3BGP-catalyzed reactions, the first-order rate constants were 0.139 ± 0.009, 0.182 ± 0.031, 0.297 ± 0.022, and 0.212 ± 0.028 for Glc, Glc-2NP, Glc-4NP, and Glc-MNP as the acceptor, respectively. With both enzymes, donor conversion rates were significantly higher with Glc-4NP as the glycosyl acceptor (*p*-values of 0.041 and 0.011 for *Ct*CDP and *Ta*1,3BGP, respectively), while reactions carried out using Glc-2NP or Glc-4NP were not significantly different from reactions carried out using Glc (Table S1). These results are in good agreement with previous reports, which suggest that Glc is a poor acceptor for *Ct*CDP and *Ta*1,3BGP.^39,48^ To our knowledge, only one previous report exists^50^ of cellodextrin phosphorylase activity in the synthesis direction with one of the acceptors used in this study, i.e., Glc-4NP. In this report, despite using a cellodextrin phosphorylase from a different organism, the authors reported slightly higher specific activity for Glc-4NP than Glc (13.6 and <1 U/mg, respectively).

Typically, synthesis reactions with cellodextrin or β-1,3-glucan phosphorylases that are carried out with a 50 mM acceptor and 200 mM donor terminate eventually at the production of an insoluble precipitate due to the low water solubility of the reaction products. In fact, efforts have been made to prevent the precipitation of cellulose oligomers during synthesis using, for example, organic solvents and ionic liquids.^51,52^ Correspondingly, cellulose synthesis reactions with *Ct*CDP resulted in the production of insoluble precipitates in all cases (Figure 2b, Table S2). The yields of insoluble product for cellulose synthesis reactions carried out with Glc, Glc-2NP, Glc-4NP, and Glc-MNP were 10.3 ± 0.4, 12.3 ± 0.4, 10.0 ± 0.1, and 14.0 ± 0.9 mg/mL, corresponding to 25 ± 1, 26 ± 1, 21 ± 0, and 28 ± 2% of theoretical maximum. Surprisingly, β-1,3-glucan synthesis reactions carried out using Glc-2NP or Glc-4NP did not result in the production of insoluble precipitate, despite high Glc-1P conversion, and with Glc-MNP, only a small amount of precipitate was formed after several days. The yields of insoluble product for reactions carried out with Glc and Glc-MNP were 21.8 ± 0.8 and 10.3 ± 1 mg/mL, corresponding to 53 ± 2 and 20 ± 0% of the theoretical maximum. These results could be an indication that the self-assembly of β-1,3-glucans (which assemble in triple-helical crystal form when synthesized using Glc as the acceptor^40^) is more easily disturbed by reducing end modifications than cellulose, which typically assembles as linear chains into cellulose_II_ crystal form.^20,26,38,39^

### Chemical Analysis of Insoluble Reaction Products

We started the characterization of the obtained insoluble precipitates by determining their molar mass distributions (MMDs) using size-exclusion chromatography (SEC) and matrix-assisted laser desorption ionization time-of-flight mass spectrometry (MALDI-ToF-MS) (Figure 2c,d, respectively). Using SEC, the number-average molar masses for celluloses ranged from 1700 to 2000 g/mol. Celluloses synthesized using Glc-2NP and Glc-4NP had the lowest molecular weight (1759 and 1768 g/mol, respectively), while synthesis with Glc or Glc-MNP as the acceptor yielded slightly higher molecular weight (1983 and 1948 g/mol, respectively). β-1,3-Glucans synthesized using Glc or Glc-MNP had higher molecular weights than celluloses at 3207 and 3222 g/mol, respectively. Similar trends could be observed with MALDI-ToF-MS, which additionally yields information on the exact molar masses of individual polymer chains. However, several unexpected peaks could be observed with MALDI-ToF-MS, suggesting possible fragmentation of the reaction products. Fragmentation of 4NP-glycosides has been reported as a problem for their analysis with MALDI-ToF-MS,^53^ possibly due to their absorption maximum close to the energy of laser light (337 nm). We wanted to further evaluate this possibility by measuring the MALDI-ToF-MS spectrum for commercially available 4-nitrophenyl α-d-maltohexaoside, which also displayed several unexpected peaks that suggested fragmentation (Figure S2). Therefore, for further chemical structure characterization, we chose to employ NMR spectroscopy.

### Chemical Structure Characterization with NMR

The precipitates produced in the reaction mixtures with Glc, Glc-2NP, Glc-4NP, and Glc-MNP were dissolved in 4% NaOD-D_2_O. Their ^1^H and ^13^C chemical shifts were assigned using standard 2D NMR techniques and are shown in Tables S3 and S4, respectively. The ^1^H NMR spectra are shown in Figure S3, and the HSQC and TOCSY spectra are shown in Figures S4–S7.

All synthesized celluloses and β-1,3-glucans exhibit signals corresponding to the H1–H6 protons of repeating β-glucosyl units at 3.2–4.4 ppm. For cellulose synthesized using glucose as the acceptor, both α- and β-anomeric proton peaks of the reducing end glucose are clearly visible at 5.3 and 4.6 ppm, and the signals of the NP group of celluloses synthesized using Glc-2NP or Glc-4NP are seen on the aromatic region at 6.5–8 ppm. However, for the products derived from Glc-MNP, the signals of the chromophore and the first glucose unit could not be assigned due to the apparent instability of the MNP unit in the employed solvent. However, NaOD is a well-studied solvent in ^1^H NMR analysis of short-chain celluloses synthesized by *Ct*CDP,^39,54,55^ as it efficiently solubilizes the reaction products. Despite the instability of the MNP group, the repeating and terminal units of the glucose chain were stable, and their NMR signals could be assigned.

In all samples, the chemical shifts and coupling constants (∼7.8 Hz) of H1 of the transferred glucose units indicate the presence of β linkages. In cellulose, cellulose-2NP, and cellulose-4NP, the C4 signals of the acceptor and midchain glucose show glycosylation shifts of about 8 ppm toward higher ppm values when compared to C4 of the terminal glucose units. This, together with HMBC couplings over the glucosidic linkages (not shown), indicates the presence of β-1,4 linked glucose chains. Similarly, the spectra of β-1,3-glucans reveal a glycosylation shift of C3 and HMBC connections over the glucosidic linkages, confirming the structure of β-1,3 linked glucose chains.

The lack or relatively minor presence of signals for Hα/Hβ peaks for cellulose synthesized using Glc-2NP, Glc-4NP, or Glc-MNP indicates that the enzymatically synthesized glucan chains retain a substitution at the C1 position of the reducing end. For Glc-2NP and Glc-4NP, ^1^H NMR signals corresponding to H3–H6 and H2–H3 of 2NP and 4NP at ∼6.4–8.0 ppm as well as HMBC couplings between glucose 1 and the chromophore further support the successful synthesis of chromophore-conjugated celluloses.

### Cellulose Structure

**Figure 3:**
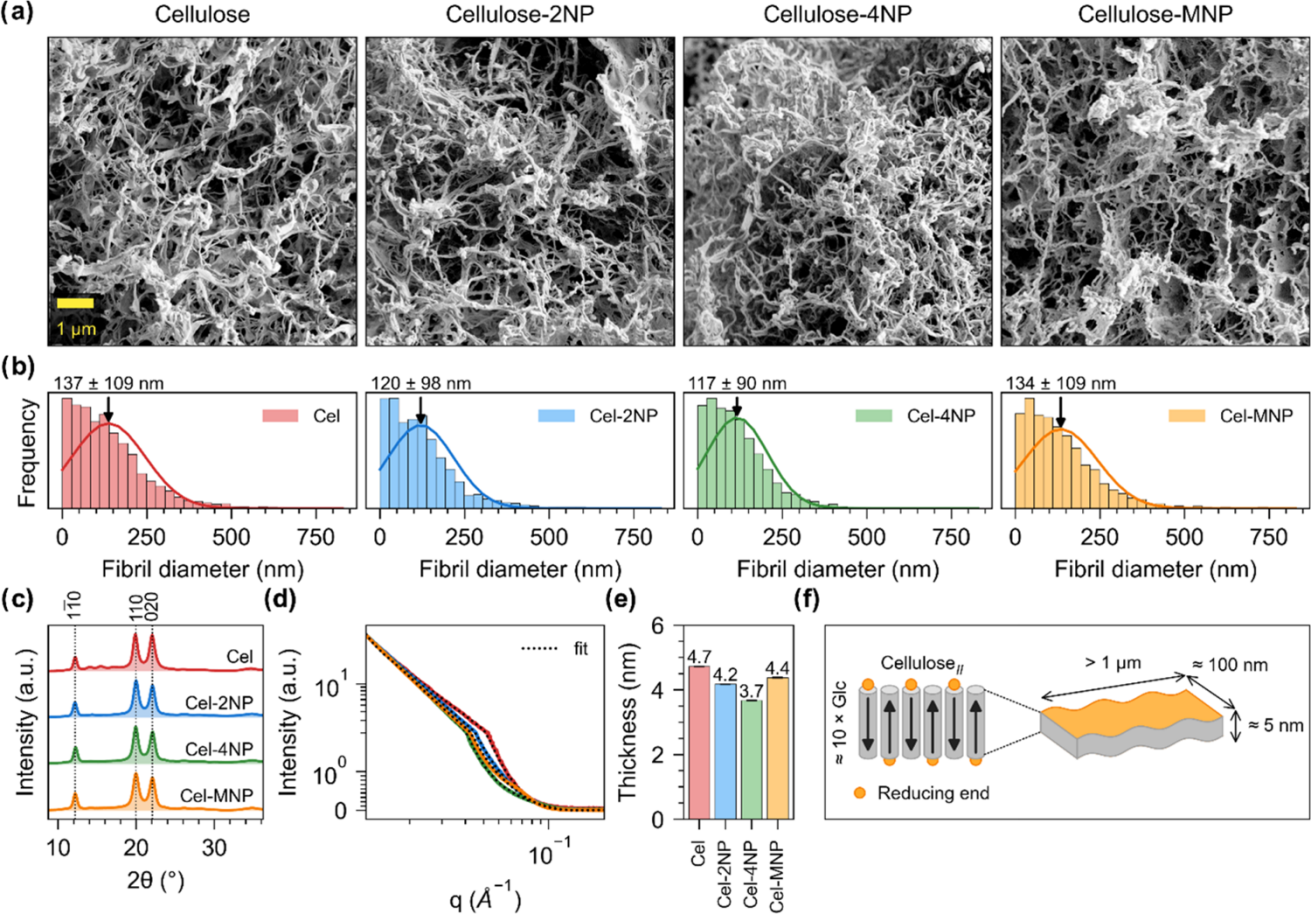
Structural characterization of enzymatically synthesized celluloses. (a) SEM images of the synthesized cellulose nanofibrils/-ribbons. The same magnification was used to obtain all of the images. (b) The calculated fibril diameter of the synthesized cellulose nanofibrils. (c) WAXS data corresponding to freeze-dried cellulose powders. (d) SAXS data corresponding to sedimented celluloses in the wet state. (e) Lamellar thickness calculated by fitting the lamellar model on SAXS data shown in (d). (f) Schematic representation of cellulose lamellae (fibril/ribbon) composed of antiparallel cellulose chains (cylinders), corresponding fibril/ribbon dimensions, and orientation of reducing ends (yellow) in cellulose lamellae.

Next, we determined the morphological characteristics and structural features of the cellulose products that were synthesized enzymatically using *Ct*CDP. Using high-resolution SEM imaging (Figure 3a, additional images in Figures S8–S11), nanofibrils/-ribbons could be observed in all cases in good agreement with previously reported structures.^35,37−39^ Quantification of fibril diameter (Figure 3b) suggested an average diameter of over 100 nm for all products, i.e., 137 ± 109, 120 ± 98, 117 ± 90, and 134 ± 109 nm when Glc, Glc-2NP, Glc-4NP, or Glc-MNP was used as the acceptor, respectively.^37,38^ According to WAXS measurements (Figure 3c), all products had cellulose_II_ crystal form, which is typical for cellulose synthesized using *Ct*CDP (cellulose Iβ crystals have been produced in only one previous study).^26,38,39^ Several authors have suggested a lamellar structural organization of enzymatically synthesized cellulose crystals, where the molecular axis of the cello-oligosaccharides is perpendicular to the plane of the crystals (Figure 3f).^39,56,57^ For this reason, we chose to estimate fibril thickness by fitting a lamellar model on the measured SAXS data (Figure 3d, detailed parameters in Table S5). This model estimated thickness values of 4.7, 4.2, 3.7, and 4.4 nm (Figure 3e) for products synthesized using Glc, Glc-2NP, Glc-4NP, or Glc-MNP as the acceptor, respectively, in good agreement with previously reported thickness of cellulose nanofibrils/-ribbons synthesized using *Ct*CDP and quantified using atomic force microscopy (AFM) or transmission electron microscopy (TEM).^21,38,39,58^ Furthermore, based on a cellobiose repeat distance of 1.04 nm,^59^ these values of thickness correspond to cellulose chains with a chain length of approximately 9.0, 8.1, 7.1, and 8.5 glucose units, respectively, and thus, have good correlation with the MMDs we obtained using SEC and MALDI-ToF-MS. Overall, these results suggest the self-assembly of *Ct*CDP synthesized cellulose chains into cellulose_II_ nanofibril networks is not significantly interfered with when chromophoric β-glycosides are used as the glycosyl acceptor. However, we found that the choice of acceptor may affect the reaction rate, chain length, fibril thickness, and to some extent fibril diameter. We have reported similar changes in cellulose_II_ fibril network morphology with different concentrations of glucose and cellobiose as the acceptor.^38^

### β-1,3-Glucan Structure

**Figure 4:**
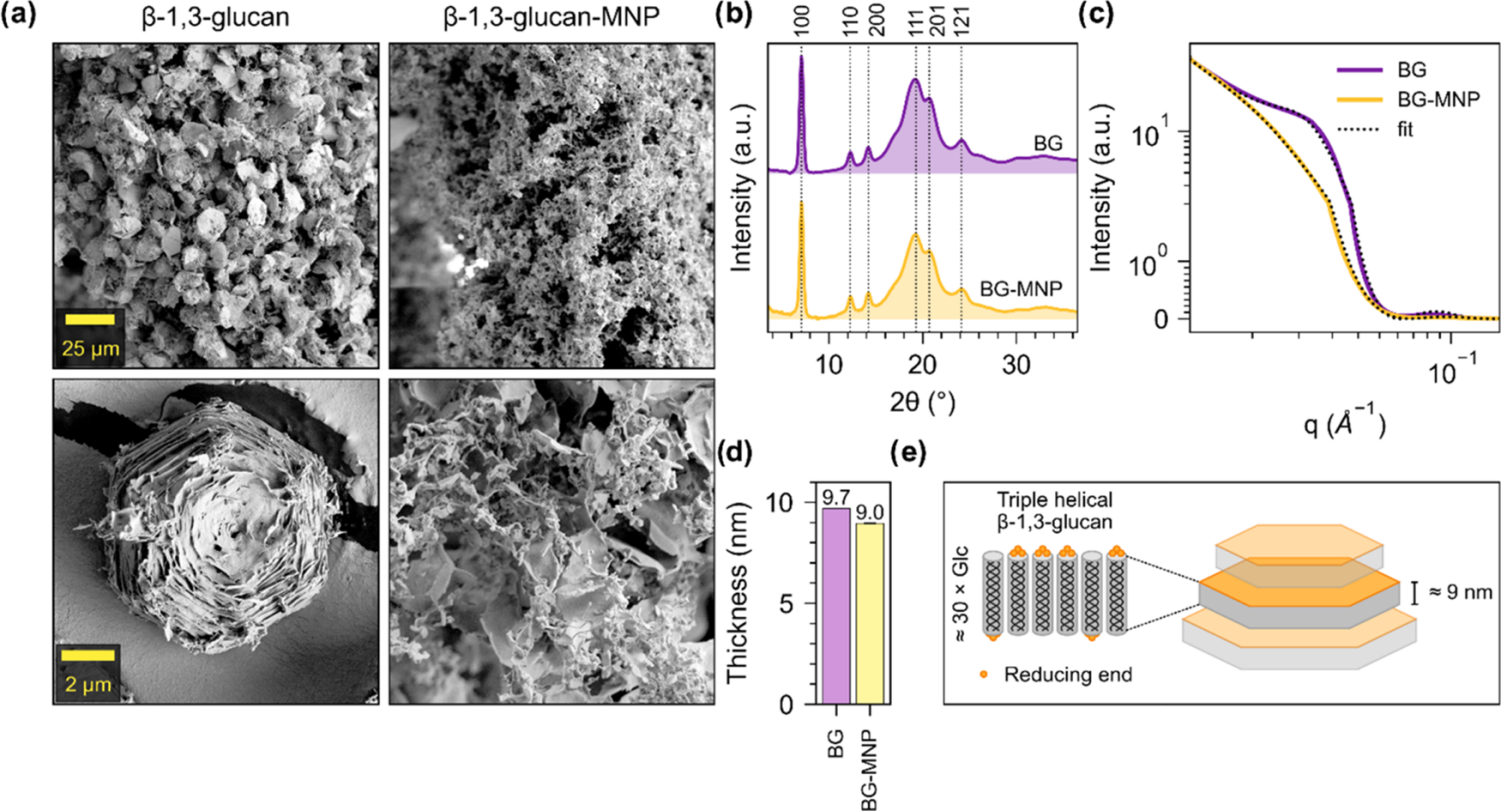
Structural characterization of enzymatically synthesized β-1,3-glucans. (a) SEM images of the synthesized β-1,3-glucans. The same magnification was used to obtain images placed horizontally. (b) WAXS data corresponding to freeze-dried β-1,3-glucan powders. (c) SAXS data corresponding to sedimented β-1,3-glucans in the wet state. (d) Lamellar thickness calculated by fitting a lamellar model on SAXS data, as shown in (c). (e) Schematic representation of β-1,3-glucan lamellae composed of β-1,3-glucan chains assembled as triple-helices (cylinders), corresponding sheet thickness, and orientation of reducing ends (yellow) in sheets.

β-1,3-Dlucan synthesis with Glc-2NP and Glc-4NP as the acceptor did not yield any insoluble product, and therefore, we structurally characterized only the crystalline products that formed when Glc or Glc-MNP were used as the acceptor. High-resolution SEM imaging of β-1,3-glucans synthesized using Glc as the acceptor (Figure 4a, additional images in Figures S12 and S13) revealed a hexagonal microparticle morphology that we have characterized previously.^40^ However, β-1,3-glucans synthesized using Glc-MNP as the acceptor did not undergo hierarchical self-assembly into well-ordered microparticles but formed less ordered structures, which resemble β-1,3-glucans synthesized at lower reaction temperatures (≤37 °C) using *Ta*1,3BGP.^40^ Based on WAXS data, both products had triple-helical crystal form (Figure 4b), as has been reported previously for β-1,3-glucans synthesized using *Ta*1,3BGP.^40^ Structural differences could be seen with SAXS, which we measured for sedimented particles in the wet state (Figure 4c, detailed parameters after fitting in Table S5). After fitting the SAXS data with the same lamellar model that we used for cellulose, we found the thicknesses of the lamellar β-1,3-glucan sheets to be 9.7 and 9.0 nm when Glc and Glc-MNP are used as the acceptor, respectively (Figure 4d). Based on a pitch of 0.2835 nm per glucose unit,^60,61^ these values of thickness correspond to chain lengths of 34.2 and 31.7 glucose units and are in agreement with the MMDs we obtained using SEC and MALDI-ToF-MS. It seems reasonable to suggest that the MNP moiety conjugated reducing ends could potentially interfere with sheet-to-sheet interactions and thus microparticle assembly, as the modified reducing ends would be situated toward the surfaces of the lamellar sheets (Figure 4e). To our knowledge, the orientation of triple-helices in these lamellar sheets is currently unclear; however, our earlier molecular dynamics simulations^40^ suggest a slightly favorable (approximately 60 kJ/mol) parallel association of two triple-helices in comparison to the antiparallel association (55 kJ/mol) and thus, we have chosen to represent them in random orientation. In a single triple-helix, the three β-1,3-glucan chains are parallel and in phase along the helix axis.^61^ In addition to structural interference, another reason β-1,3-glucans synthesized using Glc-2NP or Glc-2NP did not undergo precipitation could be the short chain length. This also seems to be a reasonable suggestion, as shorter cellulose chains were produced with these acceptors using *Ct*CDP.

### Solubilization and Regeneration

**Figure 5:**
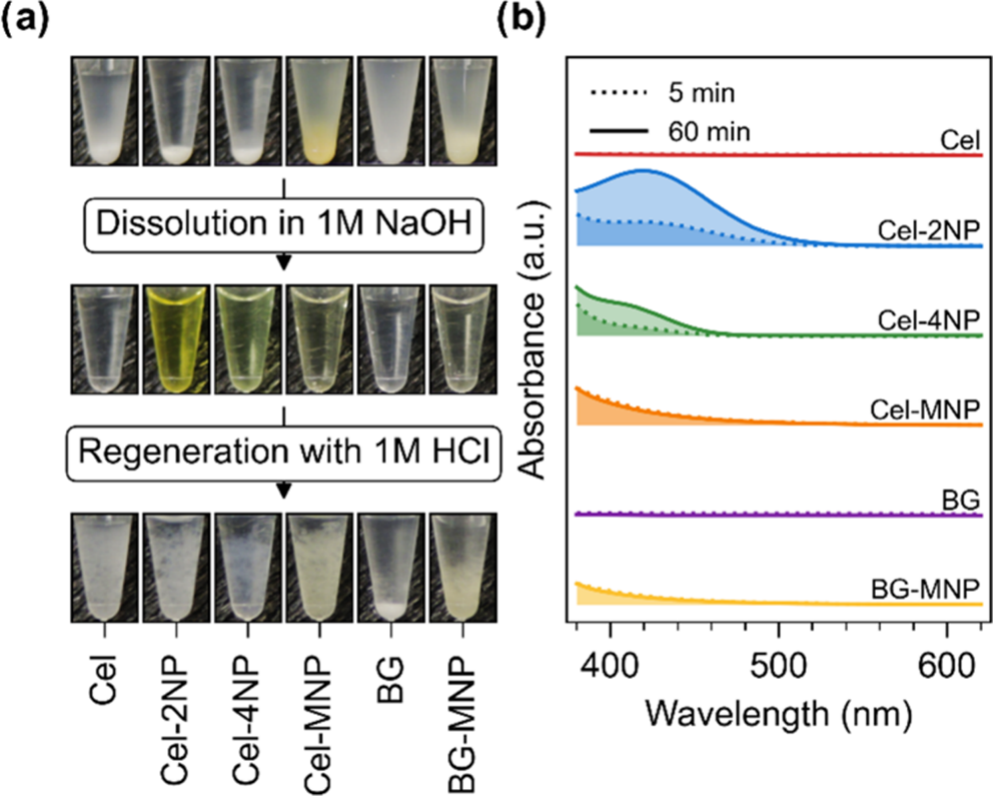
Effect of solubilization and regeneration on observed color. (a) Photographs of insoluble glucans resuspended in water, dissolved in 1 M NaOH, and regenerated using 1 M HCl. During each treatment step, the samples were diluted 1:1, resulting in concentrations of 10 mg/mL (resuspended), 5 mg/mL (dissolved), and 2.5 mg/mL (regenerated). (b) UV–vis spectra of glucans dissolved in 1 M NaOH after an incubation period of 5 and 60 min.

Because cellulose and β-1,3-glucan chains synthesized using *Ct*CDP and *Ta*1,3BGP are soluble in 1 M NaOH, and 4-nitrophenol has been reported to be yellow above pH 7.5, we also wanted to examine the color of the synthesized glucans in soluble form. Interestingly, celluloses synthesized using Glc-2NP or Glc-4NP turned yellow when solubilized in 1 M NaOH (Figure 5a), although the yellow color is commonly associated with the nitrophenolate ion.^49^ We also measured the absorbances in the visible spectrum for the solubilized polysaccharides (Figure 5b) and noted that the yellow color associated with Cel-2NP and Cel-4NP became more intense as a function of time. Neutralization of the polysaccharides with 1 M HCl yielded insoluble products with similar color as before solubilization, i.e., Cel-MNP had the most intense yellow color, followed by BG-MNP, while the rest of the products had white color. Finally, it should be noted that the synthesized 2NP- and 4NP-conjugated polysaccharides should be relatively stable in 1 M NaOH, as we did not see any major structural changes for several days when the products were analyzed with NMR using 1 M NaOD as the solvent.

## Conclusions

In this article, we investigated the feasibility of using commercially available chromophoric β-glycosides as glycosyl acceptors (Glc-2NP, Glc-4NP, Glc-MNP) for cellulose synthesis using *Ct*CDP and β-1,3-glucan synthesis using *Ta*1,3BGP. Glc-1P conversion could be measured in all cases, with similar or higher rates than with Glc, suggesting that all of the chromophoric β-glycosides are relatively good acceptors for both of the enzymes. Product precipitation could be observed in all cases with the exception of β-1,3-glucan synthesis using Glc-2NP or Glc-4NP and *Ta*1,3BGP as the enzyme, and it seems reasonable to suggest that the β-1,3-glucans remained soluble in these cases. Using SEC, we measured the MMDs of insoluble celluloses to range between 1759 and 1983 g/mol and β-1,3-glucans to range between 3207 and 3222 g/mol.

All synthesized celluloses are assembled into cellulose_II_ nanofibril/-ribbon networks with an average diameter of approximately 100 nm and an average thickness of approximately 5 nm. We noted a correlation between chain length, fibril diameter, and fibril thickness, as shorter cellulose chains assembled into thinner fibrils with a shorter diameter. The shortest chain lengths were obtained using Glc-4NP as the acceptor and the longest chain lengths were obtained using Glc. With β-1,3-glucans, the choice of acceptor had a major impact on the structure. As mentioned earlier, precipitation did not occur at all when Glc-2NP or Glc-4NP was used as the acceptor, despite high Glc-1P conversion, which suggests that these reaction products remained in soluble form. With Glc as the acceptor, β-1,3-glucans assembled into hexagonal microparticles, while using Glc-MNP as the acceptor, only less ordered structures were obtained. Based on these observations, *Ta*1,3BGP could present an interesting opportunity to generate functionalized polysaccharides in the soluble form.

In terms of the final color of the obtained insoluble products, Cel-MNP was obtained as a yellow precipitate and BG-MNP as a slightly yellow precipitate, while the rest of the products had a white appearance. The less intense yellow color of BG-MNP could be related to the ratio between reducing end groups to glucose units, as the chain length of BG-MNP was longer than that of Cel-MNP. We also looked at the color of the products in soluble form by solubilizing them in 1 M NaOH. Once solubilized, Cel-2NP had an intense yellow color and Cel-4NP a less intense yellow color, despite both appearing white in insoluble form.

These results are a useful reference for future investigations into cellulose and β-1,3-glucan synthesis using phosphorolytic enzymes and expand our knowledge of the relationship between reducing end modifications and the structure of the final products. These results could also serve as a source of inspiration for binding studies between chromophore-containing oligosaccharides and polysaccharide-based textiles or other materials (e.g., cellulose in cotton). We believe that chromophores containing celluloses in particular could be used as advanced nanomaterials, for example, as a filler to reinforce and add color to other polymeric materials. Furthermore, the transition to biotechnologically synthesized colorants could reduce the environmental footprint.

## Supporting Information Available

Individual graphs for phosphate release measurements and fitting to the first-order model; statistical comparison of donor conversion rates; yields of insoluble reaction products; MALDI-ToF-MS spectra for 4-nitrophenyl α-d-maltohexaoside; NMR spectra of insoluble reaction products; detailed SAXS data fitting parameters; and additional SEM images of insoluble products (PDF)
