# Supplementary material for: Glycoside Phosphorylase Catalyzed Cellulose and β-1,3-Glucan Synthesis Using Chromophoric Glycosyl Acceptors
Source: Biomacromolecules. 2024 Jul 18;25(8):5048–57. doi: 10.1021/acs.biomac.4c00455 (PMC11322998; doi:10.1021/acs.biomac.4c00455)
Supplement: Supplementary file 1 — bm4c00455_si_001.pdf [file bm4c00455_si_001.pdf]

Supporting information

Glycoside phosphorylase catalyzed cellulose and β-1,3-glucan synthesis using chromophoric glycosyl acceptors

Robert Pylkkänen[1,2], Hannu Maaheimo[1], Ville Liljeström[3], Pezhman Mohammadi[1], Merja Penttilä[1,2]*

[1]VTT Technical Research Centre of Finland Ltd., FI-02044 VTT, Finland

[2]Department of Bioproducts and Biosystems, School of Chemical Engineering, Aalto University, FI-00076 AALTO, Finland

[3]Nanomicroscopy Center, OtaNano, Aalto University, FI-00076 AALTO, Finland

Supplementary figures

**Figure S1**	Phosphate release measurements and fitting to first-order model	p. S2
**Table S1**	Statistical comparison of donor conversion rates	p. S2
**Table S2**	Yield of insoluble product from the synthesis reactions	p. S3
**Figure S2**	MALDI-ToF-MS spectra for 4-nitrophenyl α-D-maltohexaoside	p. S3
**Figure S3**	^{1}H NMR spectra for the synthesized products	p. S4
**Figure S4**	HSQC spectra of cellulose and cellulose-2NP	p. S5
**Figure S5**	HSQC spectra of cellulose-4NP and cellulose-MNP	p. S6
**Figure S6**	HSQC spectra of β-1,3-glucan and β-1,3-glucan-MNP	p. S7
**Figure S7**	TOCSY spectra of the synthesized products	p. S8
**Table S3**	^{1}H chemical shifts for the synthesized products	p. S9
**Table S4**	^{13}C chemical shifts for the synthesized products	p. S10
**Table S5**	Detailed parameters obtained from SAXS-data fitting.	p. S11
**Figure S8**	Additional SEM images of synthesized cellulose.	p. S12
**Figure S9**	Additional SEM images of synthesized cellulose-2NP.	p. S12
**Figure S10**	Additional SEM images of synthesized cellulose-4NP.	p. S12
**Figure S11**	Additional SEM images of synthesized cellulose-MNP.	p. S13
**Figure S12**	Additional SEM images of synthesized β-1,3-glucan.	p. S14
**Figure S13**	Additional SEM images of synthesized β-1,3-glucan-MNP.	p. S15

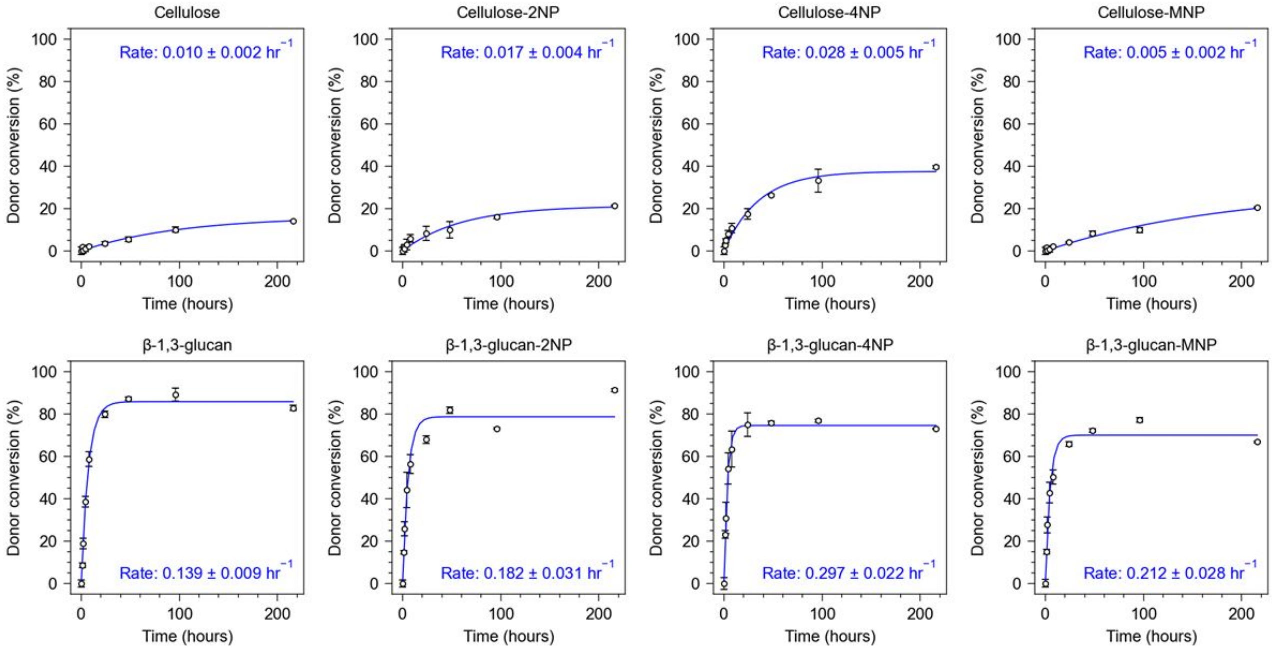

Fig. S1. Full range of experimental data for phosphate release measurements and their standard deviations (black circles and their errorbars). Result of fitting of first-order kinetics to the corresponding data (blue line). The first-order rate constant *k* and its standard deviation is given in blue text.

Product	Rate (hr^{-1})	Std. Dev. (hr^{-1})	t-stat	p-value
Cellulose	0.010	0.002	0.000	1.000
Cellulose-2NP	0.017	0.004	-2.093	0.171
Cellulose-4NP	0.028	0.005	-4.792	**0.041**
Cellulose-MNP	0.005	0.002	2.202	0.159
β-1,3-glucan	0.139	0.009	0.000	1.000
β-1,3-glucan-2NP	0.182	0.031	-1.887	0.200
β-1,3-glucan-4NP	0.297	0.022	-9.562	**0.011**
β-1,3-glucan-MNP	0.212	0.028	-3.594	0.069

Table S1. Statistical comparison of donor conversion rates between both enzymatic synthesis reactions and all four acceptors together with their corresponding t-statistic and p-value.

Product	mg/mL	%-yield
Cellulose	10.3 ± 0.4	25 ± 1
Cellulose-2NP	12.3 ± 0.4	26 ± 1
Cellulose-4NP	10.0 ± 0.1	21 ± 0
Cellulose-MNP	14.0 ± 0.9	28 ± 2
β-1,3-glucan	21.8 ± 0.8	53 ± 2
β-1,3-glucan-2NP	n.d.	n.d.
β-1,3-glucan-4NP	n.d.	n.d.
β-1,3-glucan-MNP	10.3 ± 0.1	20 ± 0

Table S2. Statistical comparison of donor conversion rates between both enzymatic synthesis reactions and all four acceptors together with their corresponding t-statistic and p-value.

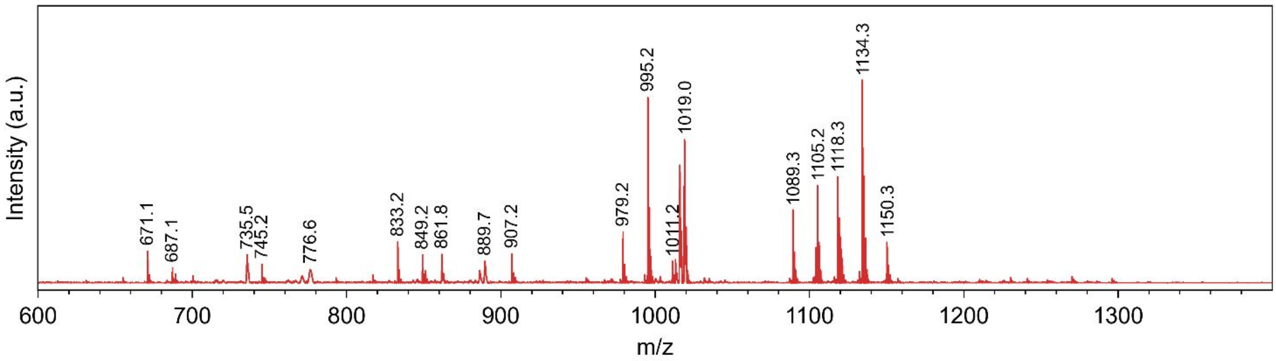

Fig. S2. MALDI-ToF-MS spectra for a commercially available 4-nitrophenyl α-D-maltohexaoside (exact mass 1111.343872) showing multiple fragmentation peaks.

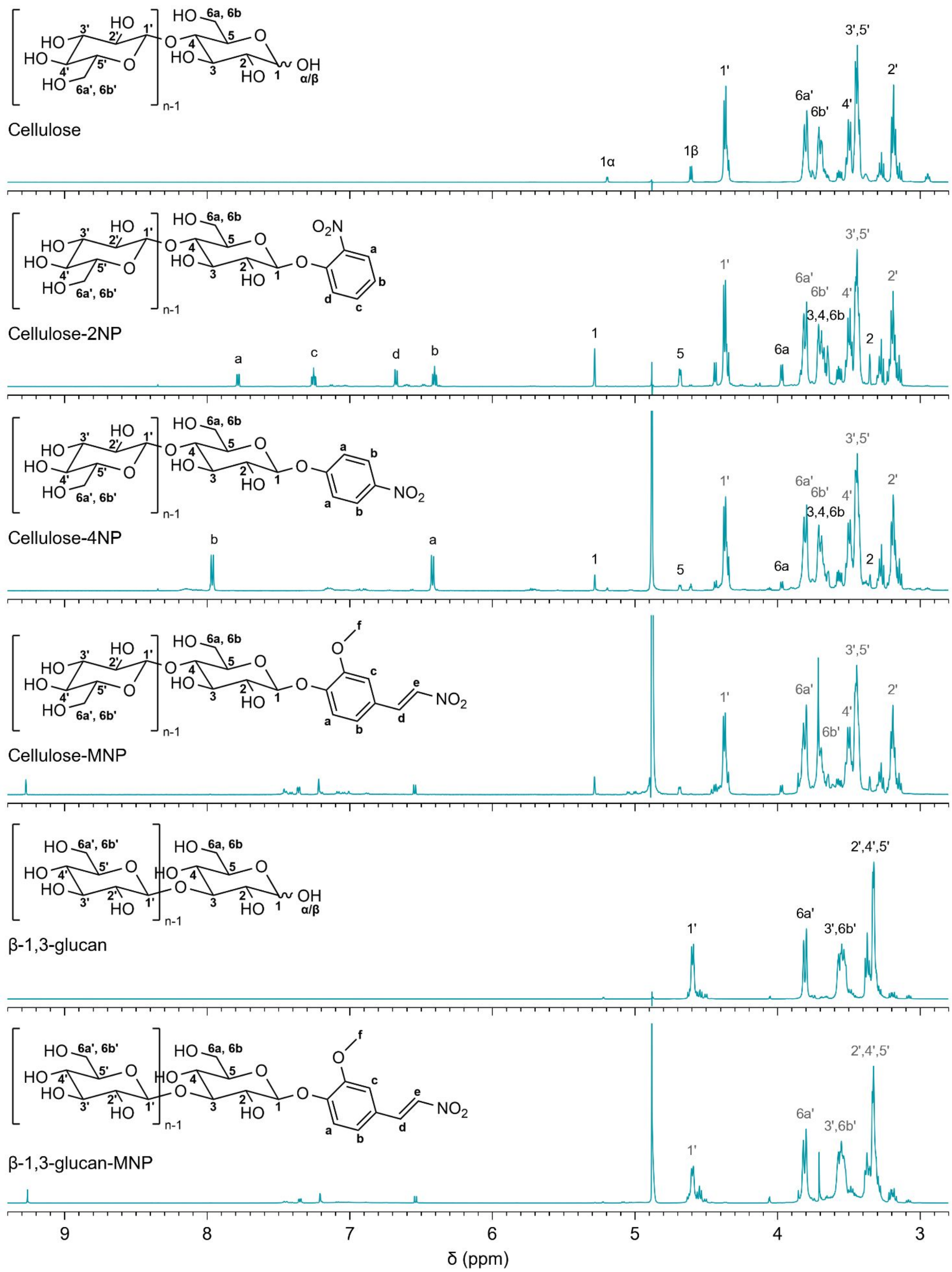

Fig. S3. ^{1}H NMR spectra for (top to bottom) cellulose, cellulose-2NP, cellulose-4NP, cellulose-MNP, β-1,3-glucan, and β-1,3-glucan-MNP dissolved in 4 % (w/w) NaOD.

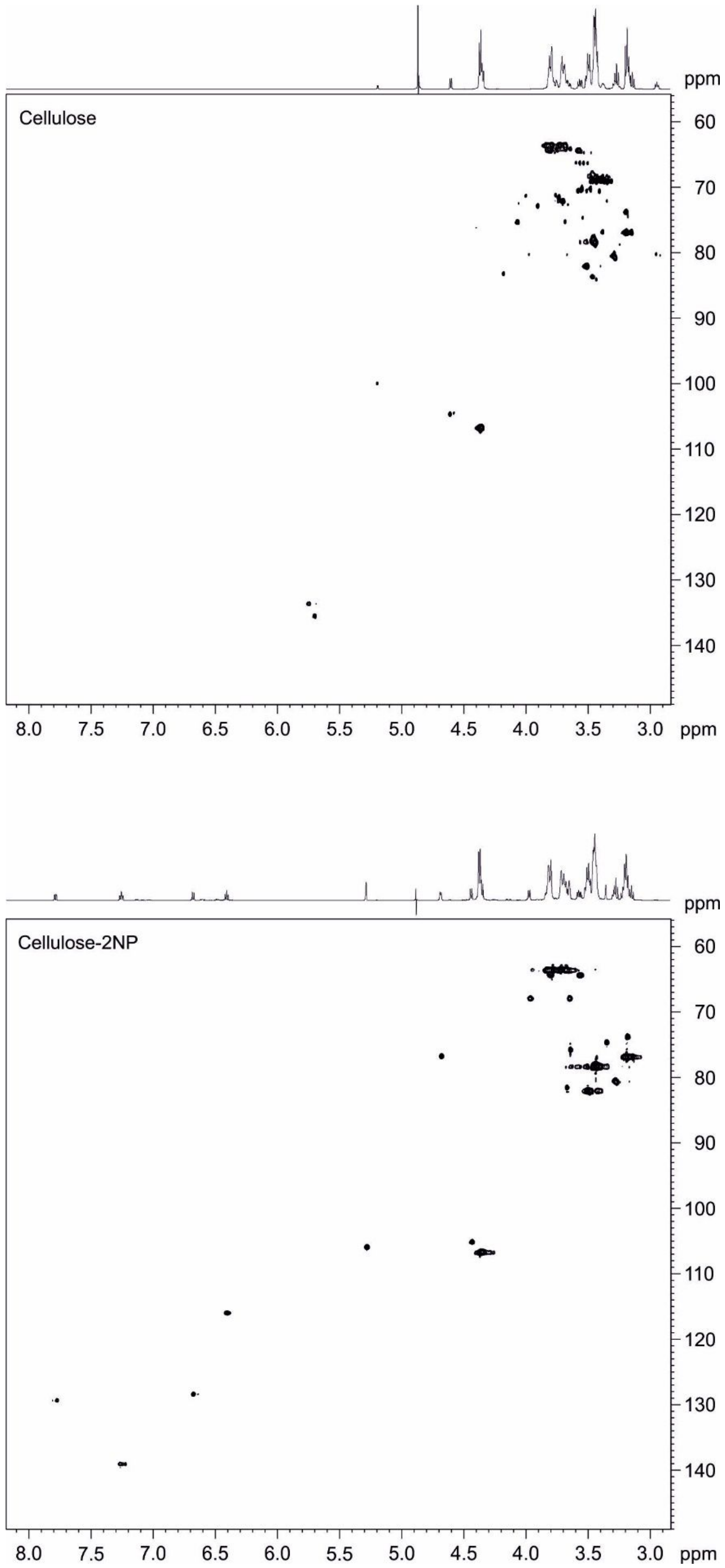

Fig. S4. Pure shift HSQC spectra of cellulose and cellulose-2NP dissolved in 4 % (w/w) NaOD.

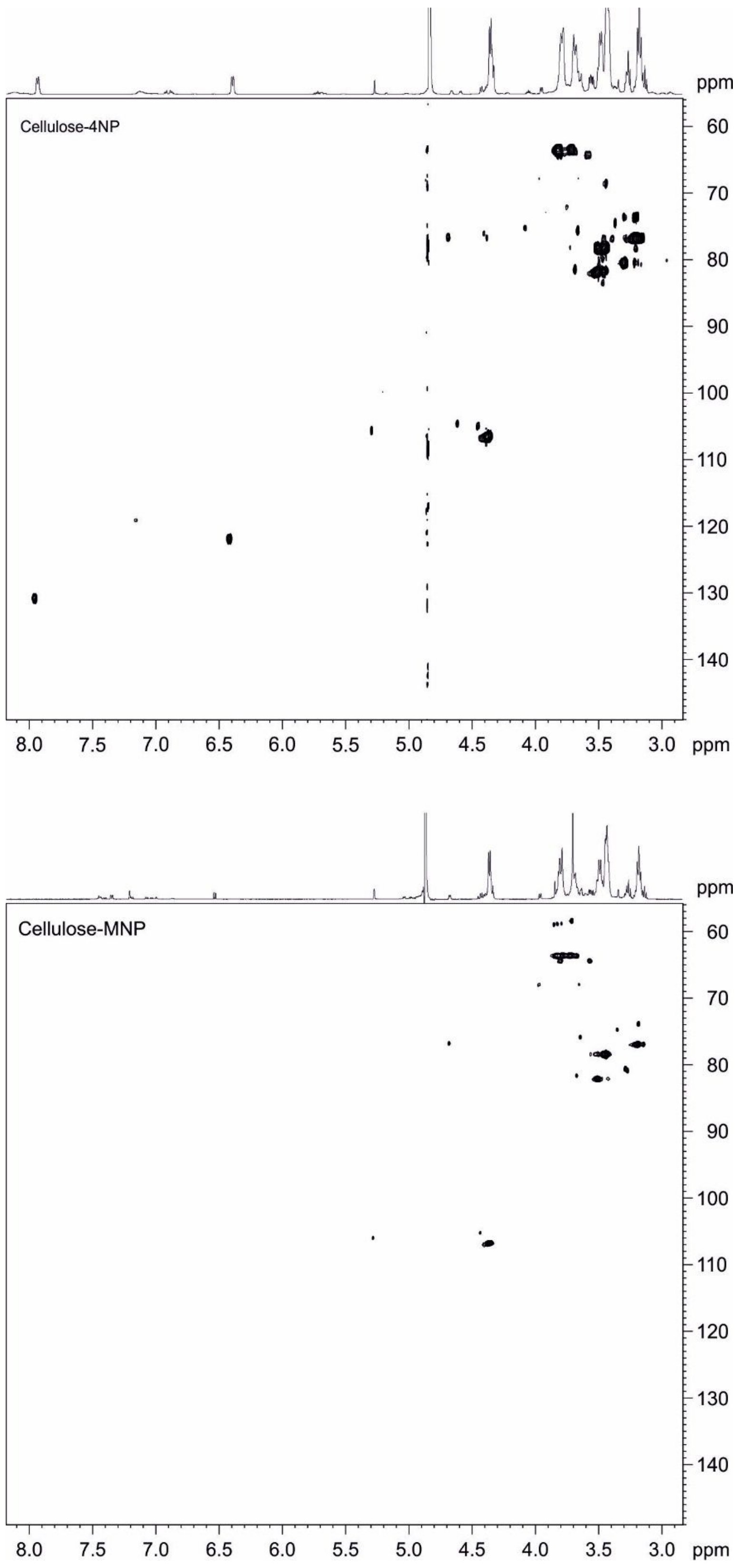

Fig. S5. HSQC spectrum of cellulose-4NP and pure shift HSQC of cellulose-MNP dissolved in 4 % (w/w) NaOD.

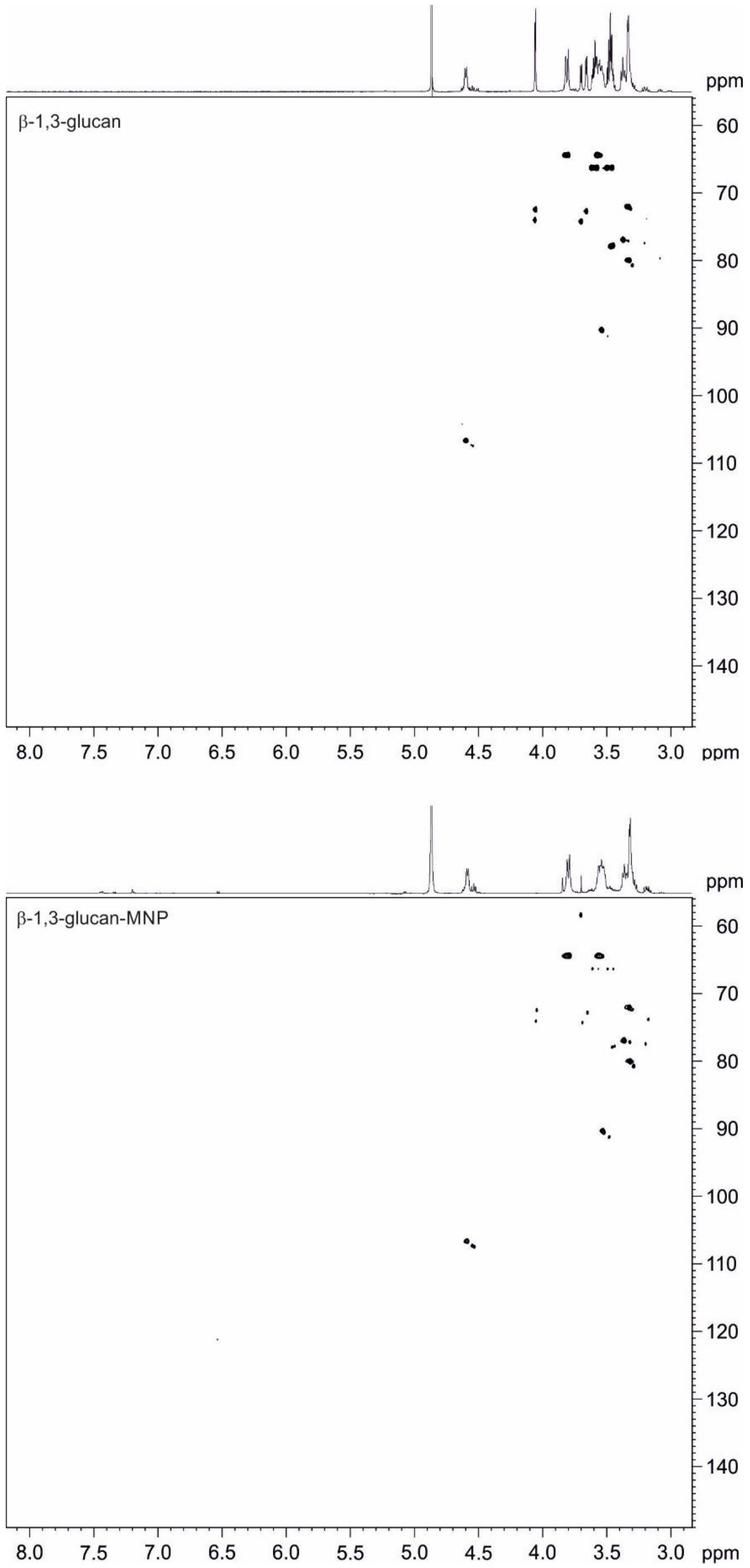

Fig. S6. Pure shift HSQC spectra of β-1,3-glucan and β-1,3-glucan-MNP dissolved in 4 % (w/w) NaOD.

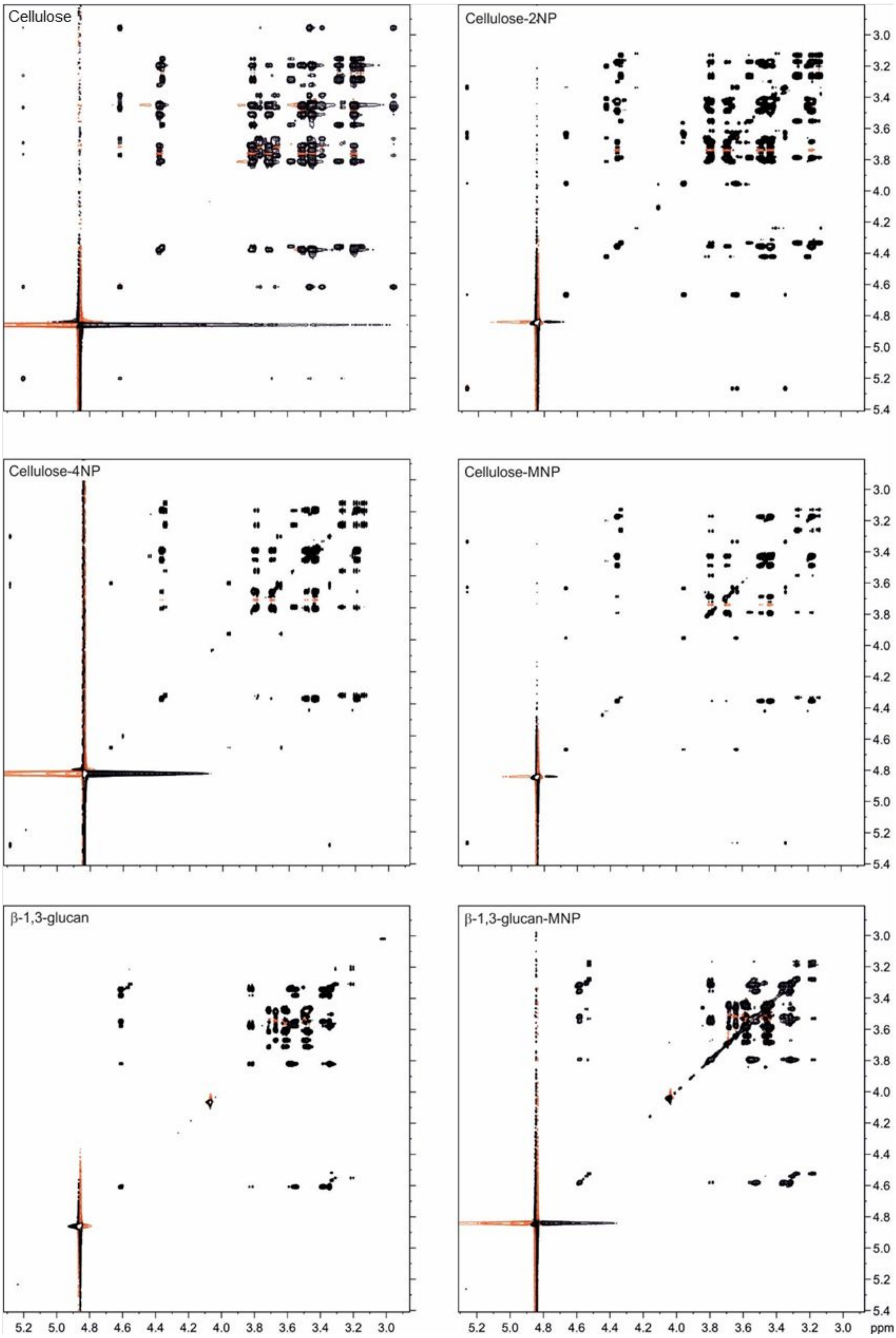

Fig. S7. Expansions of TOCSY spectra to the carbohydrate are of cellulose, cellulose-2NP, cellulose-4NP, cellulose-MNP, β-1,3-glucan and β-1,3-glucan-MNP dissolved in 4 % (w/w) NaOD.

Table S3. ^{1}H chemical shifts of cellulose, cellulose-2NP, cellulose-4NP, cellulose-MNP, β-1,3-glucan-MNP, glucose-2NP and glucose-4NP at 22 °C in 4% NaOD in D_2O. The chemical shifts were referenced to external TSP in D_2O.

Residue	Proton	Chemical shift in compound						
		Cel (α/β)	Cel-2NP	Cel-4NP	Cel-MNP	BG-MNP	Glc-2NP	Glc-4NP
Glc_1	H1	5.195/4.608	5.282	5.281	n.d.	n.d.	5.263	5.272
	H2	3.260/2.949	3.352	3.354	n.d.	n.d.	3.322	3.331
	H3	3.689/3.459	3.664	3.665	n.d.	n.d.	3.493	3.504
	H4	3.431/3.466	3.674	3.670	n.d.	n.d.	3.469	3.480
	H5	3.765/3.384	4.682	4.673	n.d.	n.d.	4.440	4.450
	H6a	3.782/3.773	3.969	3.990	n.d.	n.d.	3.954	3.964
	H6b	3.714/3.663	3.650	3.672	n.d.	n.d.	3.608	3.617
Glc_2	H1	4.372	4.436	4.446	n.d.	n.d.	-	-
	H2	3.190	3.216	3.223	n.d.	n.d.	-	-
	H3	3.44	3.43	3.43	n.d.	n.d.	-	-
	H4	3.513	3.480	3.479	n.d.	n.d.	-	-
	H5	3.44	3.43	3.43	n.d.	n.d.	-	-
	H6a	3.813	3.825	3.827	n.d.	n.d.	-	-
	H6b	3.704	3.673	3.673	n.d.	n.d.	-	-
Glc_n	H1	4.372	4.370	4.370	4.365	4.602	-	-
	H2	3.190	3.189	3.188	3.187	3.378	-	-
	H3	3.44	3.439	3.44	3.44	3.542	-	-
	H4	3.513	3.505	3.504	3.501	3.337	-	-
	H5	3.44	3.439	3.44	3.44	3.331	-	-
	H6a	3.813	3.805	3.807	3.802	3.806	-	-
	H6b	3.704	3.701	3.698	3.699	3.577	-	-
Glc_{term}	H1	4.348	4.348	4.347	4.342	4.550	-	-
	H2	3.149	3.146	3.143	3.157	3.208	-	-
	H3	3.278	3.272	3.270	3.281	3.302	-	-
	H4	3.190	3.186	3.183	3.194	3.184	-	-
	H5	3.290	3.287	3.283	3.285	n.d.	-	-
	H6a	3.805	3.800	3.797	3.812	3.822	-	-
	H6b	3.572	3.568	3.565	3.579	3.56	-	-
2NP	Ha	-	7.783	-	-	-	7.777	-
	Hb	-	6.405	-	-	-	6.400	-
	Hc	-	7.252	-	-	-	7.249	-
	Hd	-	6.674	-	-	-	6.675	-
4NP	Ha	-	-	6.418	-	-	-	6.417
	Hb	-	-	7.953	-	-	-	7.953

Table S4. ^{13}C chemical shifts of cellulose, cellulose-2NP, cellulose-4NP, cellulose-MNP, β-1,3-glucan-MNP, glucose-2NP and glucose-4NP at 22 °C in 4% NaOD in D_2O. The chemical shifts were referenced to external TSP in D_2O.

Residue	Carbon	Chemical shift in compound						
		Cel (α/β)	Cel-2NP	Cel-4NP	Cel-MNP	BG-MNP	Glc-2NP	Glc-4NP
Glc_1	C1	99.9/104.6	105.8	105.7	n.d.	n.d.	105.8	105.6
	C2	77.2/80.1	74.5	74.3	n.d.	n.d.	74.5	74.5
	C3	75.1/77.5	75.6	75.6	n.d.	n.d.	77.5	77.1
	C4	83.9/83.6	81.4	81.4	n.d.	n.d.	74.9	74.7
	C5	71.0/76.7	76.6	76.6	n.d.	n.d.	80.1	79.9
	C6	63.4/64.0	67.8	67.7	n.d.	n.d.	68.1	67.9
Glc_2	C1	106.7	105.0	n.d.	n.d.	n.d.	-	-
	C2	76.7	76.9	n.d.	n.d.	n.d.	-	-
	C3	78.2	78.4	n.d.	n.d.	n.d.	-	-
	C4	81.9	82.2	n.d.	n.d.	n.d.	-	-
	C5	78.2	78.1	n.d.	n.d.	n.d.	-	-
	C6	63.4	63.6	n.d.	n.d.	n.d.	-	-
Glc_n	C1	106.7	104.9	106.7	106.5	106.5	-	-
	C2	76.7	76.7	76.7	76.6	76.9	-	-
	C3	78.2	78.4	78.2	78.2	90.2	-	-
	C4	81.9	81.9	81.9	81.8	80.5	-	-
	C5	78.2	78.1	78.2	78.2	79.9	-	-
	C6	63.4	63.4	63.4	63.4	64.2	-	-
Glc_{term}	C1	106.5	106.5	106.5	106.4	107.3	-	-
	C2	76.7	76.7	76.7	73.6	77.3	-	-
	C3	80.7	80.6	80.6	80.7	80.5	-	-
	C4	73.6	73.7	73.7	73.6	73.7	-	-
	C5	80.4	80.4	80.4	80.4	n.d.	-	-
	C6	64.3	64.2	64.2	64.2	64.2	-	-
2NP	Ca	-	129.2	-	-	-	129.4	-
	Cb	-	115.9	-	-	-	116.0	-
	Cc	-	139.0	-	-	-	139.2	-
	Cd	-	128.3	-	-	-	128.5	-
4NP	Ca	-	-	119.1	-	-	-	121.7
	Cb	-	-	129.0	-	-	-	130.8

Table S5. Parameters obtained from SAXS-data fitting of a lamellar paracrystal model on the measured data.

Sample	Cellulose	Cellulose-2NP	Cellulose-4NP	Cellulose-MNP	β-1,3-glucan	β-1,3-glucan-MNP
Lamellar sheet thickness (Å)	47.2 ± 0.2	41.7 ± 0.1	36.7 ± 0.1	43.8 ± 0.2	97.1 ± 0.2	89.6 ± 0.2
Number of layers	20.3 ± 1.2	25.7 ± 0.7	16.3 ± 0.2	10.4 ± 0.1	5.7 ± 0.1	1.1 ± 0.0
Lamellar spacing of paracrystal stack (Å)	66.4 ± 0.6	45.9 ± 0.3	32.2 ± 0.3	46.7 ± 0.4	170.5 ± 1.1	306.3 ± 5.2
Sigma (polydispersity) of the lamellar spacing	0.88 ± 0.01	1.03 ± 0.01	1.18 ± 0.01	0.81 ± 0.01	0.38 ± 0.00	0.16 ± 0.03
Layer scattering length density (10-6 Å)	258 ± 15969081	259 ± 10876915	58 ± 3059534	253 ± 38414488	211 ± 66536682	261 ± 48677498
Solvent scattering length density (10-6 Å)	258 ± 15971240	259 ± 10877159	58 ± 3059634	253 ± 38430184	210 ± 66525852	261 ± 48686789
Scale factor or volume fraction	233 ± 3233108	226 ± 37153905	573 ± 54897	232 ± 118890572	215 ± 197090246	233 ± 137024063
Source background (cm^{-1})	0.0003 ± 0.0000	0.0007 ± 0.0000	0.0000 ± 0.0000	0.0035 ± 0.0003	-0.0017 ± 0.0006	0.0005 ± 0.0001
R^2	0.99991	0.99999	0.99995	0.99889	0.99815	0.99971

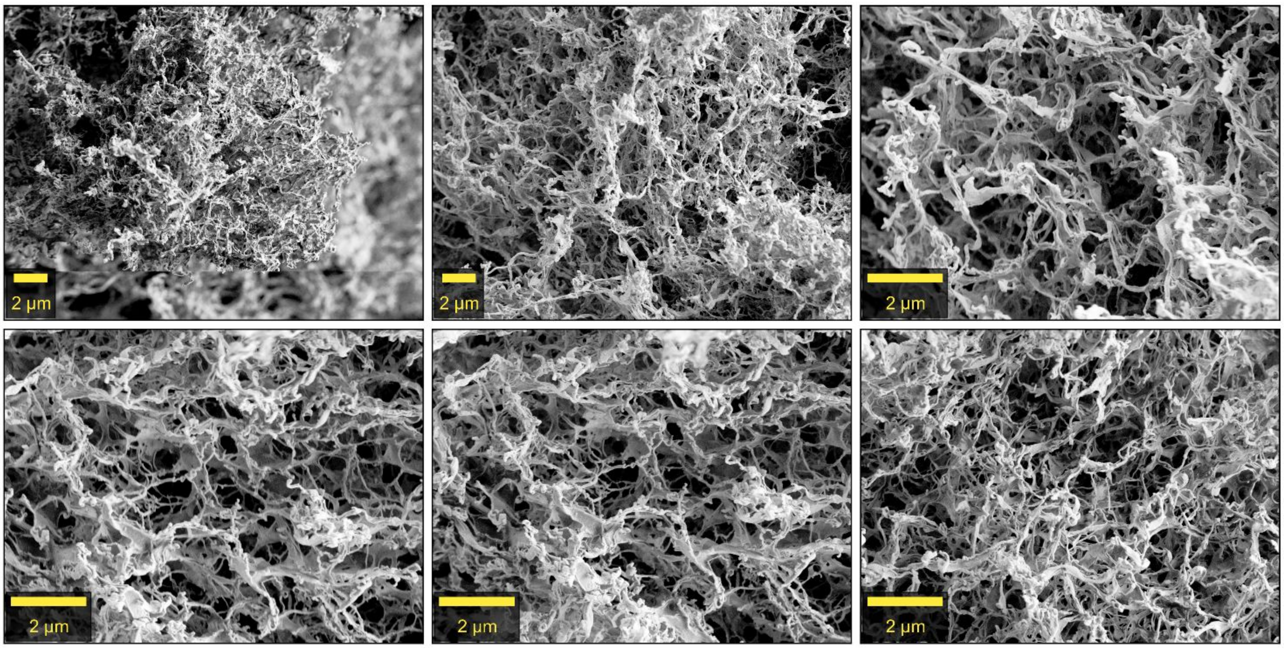

Fig. S8. Additional SEM images of synthesized cellulose.

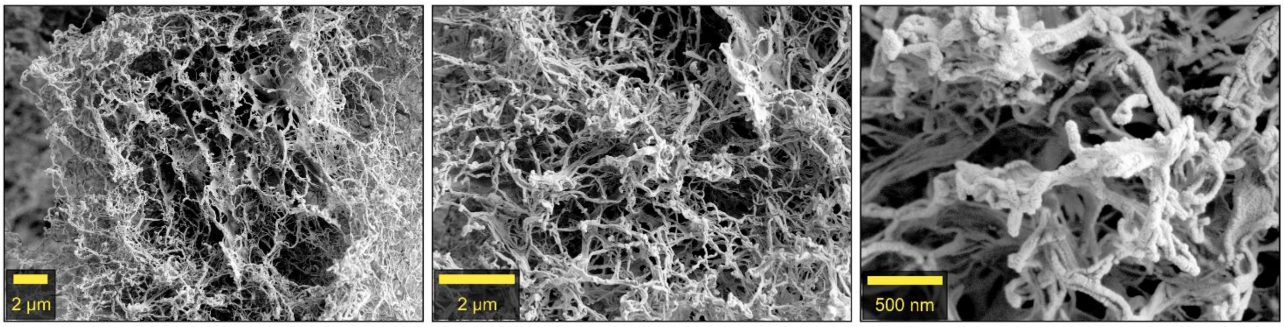

Fig. S9. Additional SEM images of synthesized cellulose-2NP.

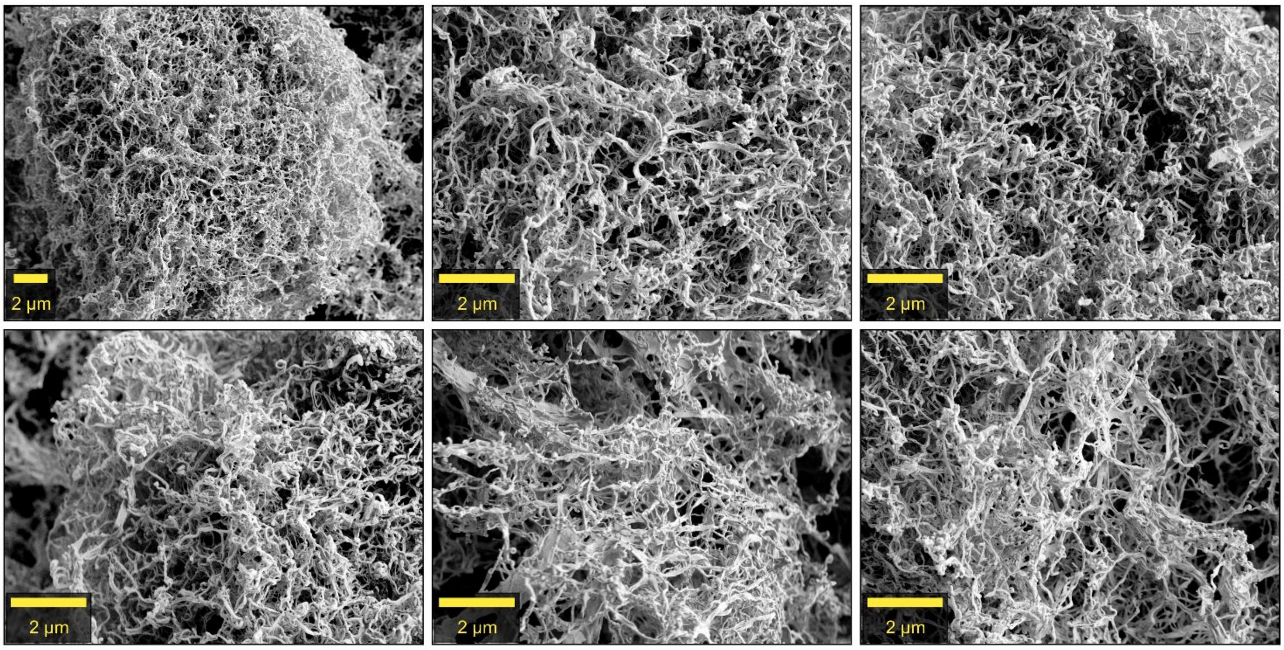

Fig. S10. Additional SEM images of synthesized cellulose-4NP.

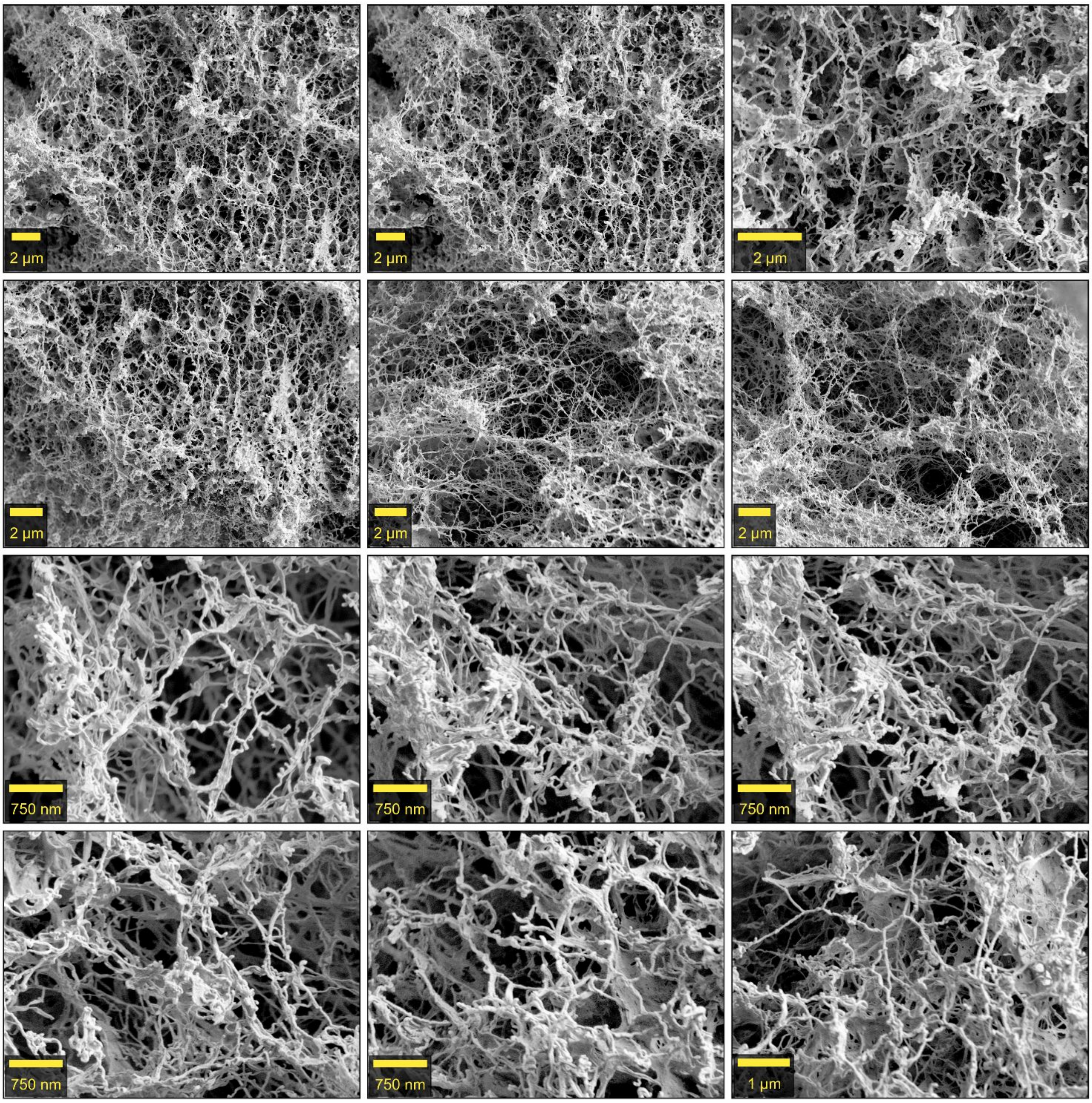

Fig. S11. Additional SEM images of synthesized cellulose-MNP.

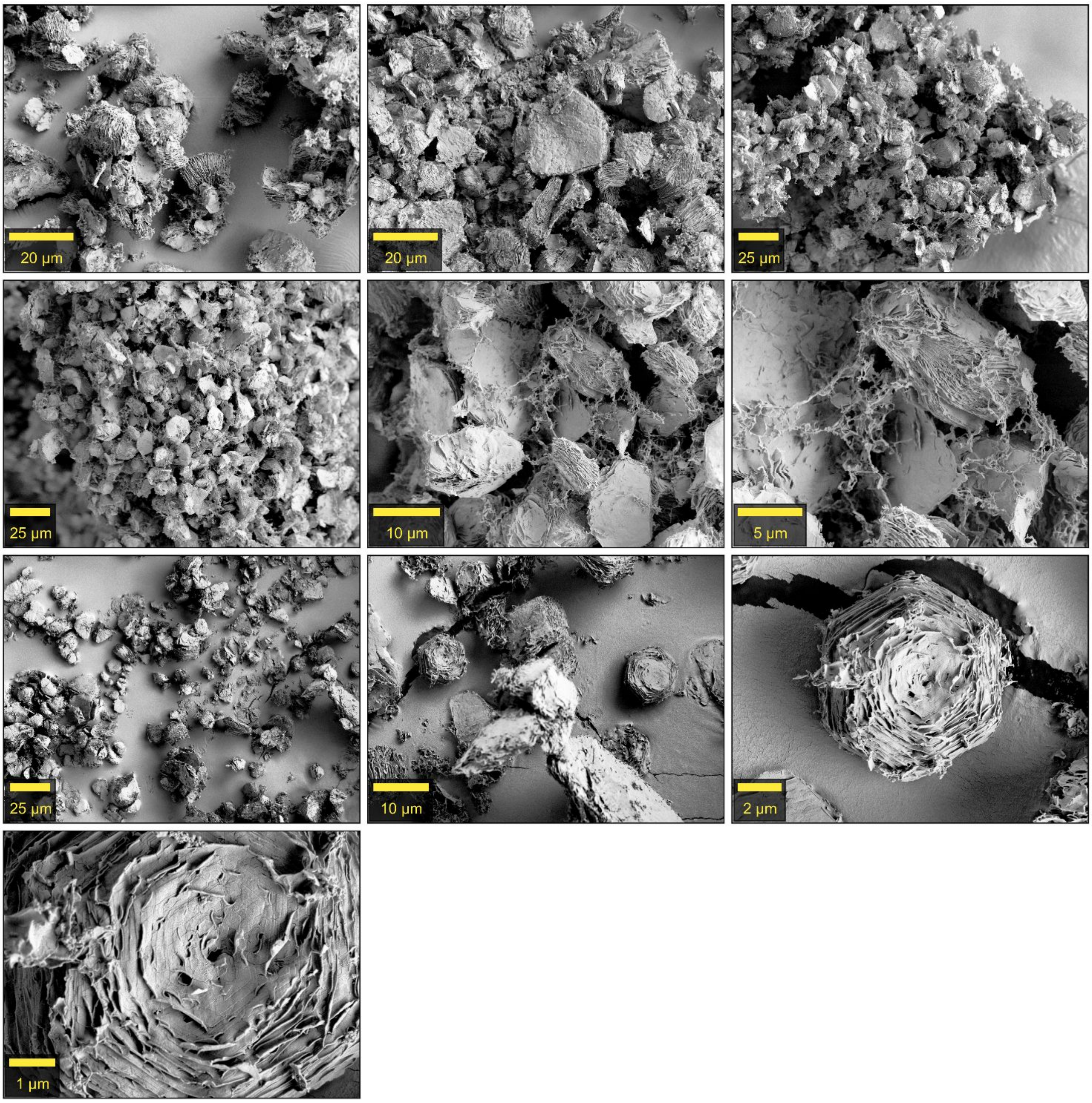

Fig. S12. Additional SEM images of synthesized β-1,3-glucan.

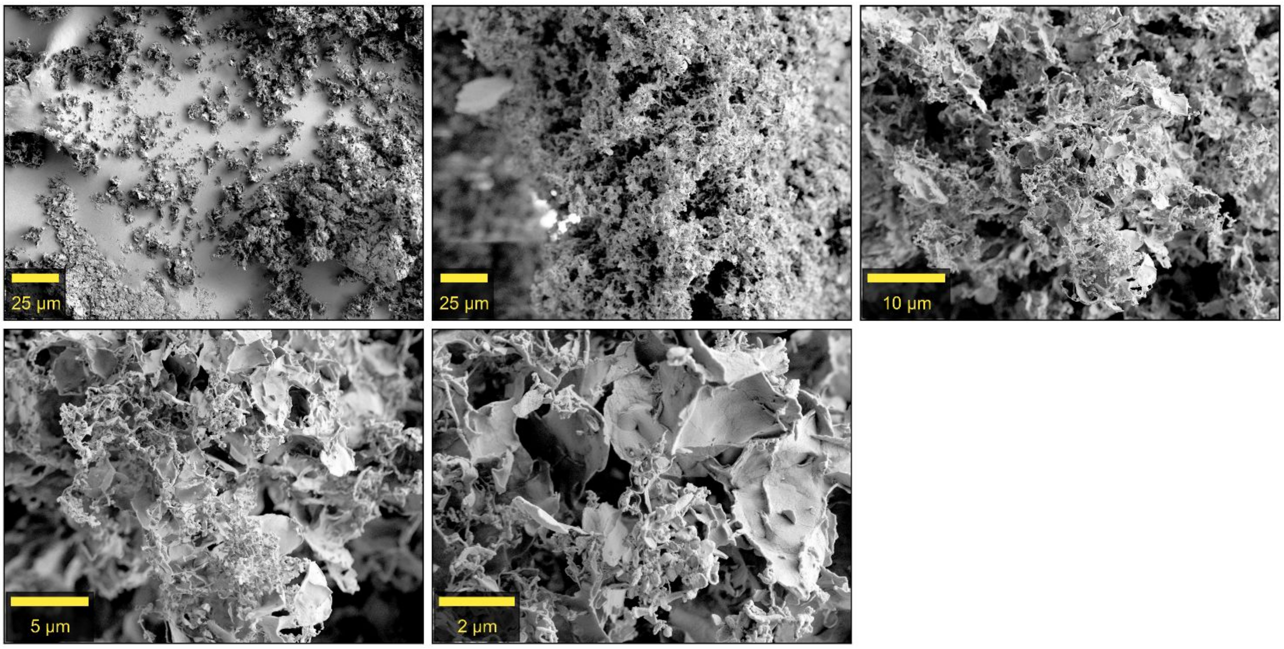

Fig. S13. Additional SEM images of synthesized β-1,3-glucan-MNP.
